# Regulatory mechanisms of testosterone-stimulated song in the sensorimotor nucleus HVC of female songbirds

**DOI:** 10.1186/s12868-014-0128-0

**Published:** 2014-12-02

**Authors:** Falk Dittrich, Claudia Ramenda, Doris Grillitsch, Carolina Frankl-Vilches, Meng-Ching Ko, Moritz Hertel, Wolfgang Goymann, Andries ter Maat, Manfred Gahr

**Affiliations:** Max Planck Institute for Ornithology, Department of Behavioural Neurobiology, Eberhard-Gwinner Strasse, Haus 6a, Seewiesen, 82319 Germany

**Keywords:** Songbird, Soft song, European robin, Canary, Microarray, Transcriptome, Testosterone, Song control nucleus, HVC

## Abstract

**Background:**

In male birds, influence of the sex steroid hormone testosterone and its estrogenic metabolites on seasonal song behavior has been demonstrated for many species. In contrast, female song was only recently recognized to be widespread among songbird species, and to date, sex hormone effects on singing and brain regions controlling song development and production (song control nuclei) have been studied in females almost exclusively using domesticated canaries (*Serinus canaria*). However, domesticated female canaries hardly sing at all in normal circumstances and exhibit only very weak, if any, song seasonally under the natural photoperiod. By contrast, adult female European robins (*Erithacus rubecula*) routinely sing during the winter season, a time when they defend feeding territories and show elevated circulating testosterone levels. We therefore used wild female European robins captured in the fall to examine the effects of testosterone administration on song as well as on the anatomy and the transcriptome of the song control nucleus HVC (*sic*). The results obtained from female robins were compared to outcomes of a similar experiment done in female domesticated canaries.

**Results:**

Testosterone treatment induced abundant song in female robins. Examination of HVC transcriptomes and histological analyses of song control nuclei showed testosterone-induced differentiation processes related to neuron growth and spacing, angiogenesis and neuron projection morphogenesis. Similar effects were found in female canaries treated with testosterone. In contrast, the expression of genes related to synaptic transmission was not enhanced in the HVC of testosterone treated female robins but was strongly up-regulated in female canaries. A comparison of the testosterone-stimulated transcriptomes indicated that brain-derived neurotrophic factor (BDNF) likely functions as a common mediator of the testosterone effects in HVC.

**Conclusions:**

Testosterone-induced singing of female robins correlated with cellular differentiation processes in the HVC that were partially similar to those seen in the HVC of testosterone-treated female canaries. Other modes of testosterone action, notably related to synaptic transmission, appeared to be regulated in a more species-specific manner in the female HVC. Divergent effects of testosterone on the HVC of different species might be related to differences between species in regulatory mechanisms of the singing behavior.

**Electronic supplementary material:**

The online version of this article (doi:10.1186/s12868-014-0128-0) contains supplementary material, which is available to authorized users.

## Background

Seasonal changes in bird song are mediated by testosterone and associated with neuroanatomical modifications in the song control system [1], a network of forebrain song control nuclei [2]. Both song control nuclei HVC (*sic*) and RA (robust nucleus of the arcopallium) express the androgen receptor [3]. These nuclei constitute the motor pathway of the song control system that controls song production [4-6]. The HVC is involved in the control of stereotyped song timing [7,8] and processing of syntactic information [9]. The anterior forebrain pathway that is necessary for sensorimotor song learning [10] includes a second population of HVC projection neurons that innervate the basal ganglia-like Area X (proper name) and feed back on RA via two further brain areas.

Female song is a behavior particularly common in songbird species from tropical latitudes, but there is increasing evidence that it also occurs in bird species inhabiting temperate zones [11-14]. In some songbird species such as the canary [15-25], chaffinch [26,27], blackbird [28], Oregon and Mexican Juncos [29], white-crowned sparrow [30-32], yellowhammer [33] and song sparrow [34], adult female singing occurs rarely but testosterone has been shown to stimulate singing activity. Likewise, in female starlings that sing at high rates while having low plasma testosterone levels [35] song rate increases in response to testosterone administration [36-39]. However, there is no evidence that natural circulating testosterone levels are involved in regulating female song for these species. In contrast, female European robins display robust song when they defend feeding territories in winter, during which they also show elevated plasma testosterone levels [40]. Female European robins are therefore an excellent model species with which to study cellular processes in the song control nuclei that parallel testosterone-induced song development. Since female robin song development is related to establishing winter territories the anatomical and molecular events underlying song development are difficult to study in the field. For this study we caught female robins in the fall and mimicked experimentally high levels of testosterone in a controlled setting.

In European robins, only males express high-amplitude full songs, both for courtship and territory defense, during the breeding season whilst both sexes may use high-amplitude full song to defend their feeding territories in autumn and winter [41-45]. Full song is characterized by harmonic overtones and syllables with frequencies above 15 kHz. Low-amplitude songs are produced by both sexes as continuous streams of notes [42,44] that we will refer to as “soft song”. Female robins normally produce soft songs only in autumn and not during the breeding season [44].

So far, the cellular and molecular mechanisms involved in testosterone-dependent seasonal changes in the female song control system have been predominantly studied in canaries. Testosterone treatment increases the volumes of HVC, RA, MAN and Area X in adult female canaries [19,25,46-50], most likely by stimulating neuronal processes including cell spacing and growth of somata and dendrites, as well as increasing the number of HVC neurons and glia cells [19,20,50-55]. Testosterone-induced angiogenesis in the HVC of female canaries [56] is associated with an up-regulation of the vascular endothelial growth factor (VEGF), the VEGF receptor kinase insert domain receptor (KDR) and brain-derived neurotrophic factor (BDNF) expression [57]. Although the blocking of VEGF receptor activity does not inhibit a testosterone-induced increase in HVC volumes it does prevent androgenic induction of song development in adult female canaries [58]. The up-regulation of BDNF expression in endothelial cells of the HVC supports testosterone-dependent recruitment and survival of new neurons into HVC [57,59]. Furthermore, an effect of sex steroid hormones on the recruitment and survival of new neurons in the HVC was also found in the adult female European starling [60].

In spring, adult female European robins have low endogenous plasma testosterone levels [61] and usually do not sing [42]. However, female robins start singing once testosterone levels are elevated following treatment with testosterone [61] or naturally in autumn and winter [42]. Here, we studied female robins that were caught in the fall, when HVC was expected to develop testosterone responsiveness, to gain information about cellular and molecular effects of testosterone on female song control nuclei. We supported our results on a genome-wide gene expression analysis of the HVC by conducting immunofluorescence and in-situ hybridization experiments. Further, we compared the testosterone-induced changes of the European robin HVC transcriptome with the testosterone effect on the HVC transcriptome of the female canary. In the female canary, one testosterone-stimulated gene in the HVC, the neurotrophin BDNF, affects cell recruitment and survival [57] and is required for the development of male-like high song pattern such as syllable numbers [58] or number of high-speed tours [62]. Thus, in addition to a general analysis of gene networks we focused in particular on testosterone-induced changes in BDNF expression and in potential BDNF-driven gene networks in the HVC.

## Results

### Increased song in females

**Figure 1:**
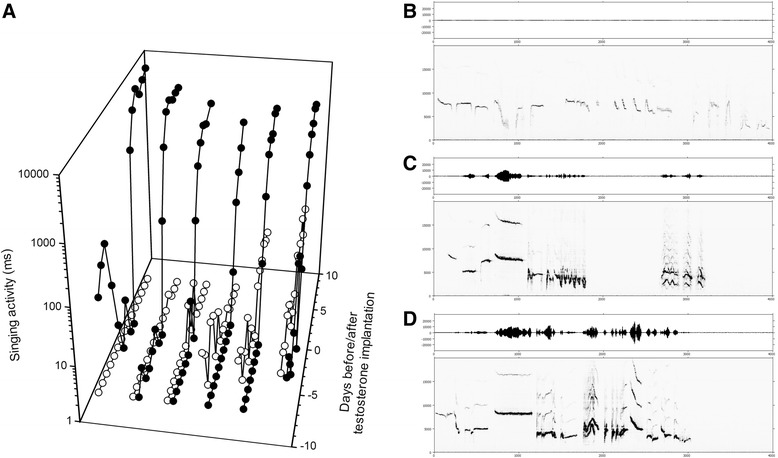
**Testosterone stimulated the production of soft song in female European robins. (A)** Daily singing activity was recorded from testosterone (●) and time-matched control (○) birds. Testosterone pellets were subcutaneously implanted at day zero. **(B-D)** Sound waves (amplitude (analog/digital arbitrary units) over time (ms)) are shown in the upper panels and sonograms are presented in the lower panels (frequency (Hz) over time (ms)). For female robin # 14 **(B)** details of soft song after seven days of testosterone treatment and **(C)** first high amplitude song phrases after eleven days are depicted. In **(D)**, a small portion of full song obtained from an adult male robin is depicted.

In female robins testosterone treatment first stimulated the production of soft song: while daily soft song rate was low in the week before testosterone pellet implantation (mean ± SEM, both groups combined: 0.3 ± 0.1 min) and did not differ between the control and testosterone group (Kruskal-Wallis Test; n = 6, p = 0.872), five out of six testosterone-treated female robins produced streams of soft notes beginning two days after implantation. By the third day of testosterone treatment daily soft song production had increased in testosterone-treated robins (7.6 ± 11.9 min) as compared to controls (0.2 ± 0.1 min) (p <0.01; residual maximum likelihood (REML) method with period of treatment as random factor followed by Tukey HSD with α = 0.05 for effect of treatment) (Figure 1A). The increase in song activity resulted in the development of a continuous stream of soft notes (Figure 1B). Mean song pitch increased significantly between days three and seven (mean ± SEM: 2420 ± 82 Hz for controls and 2735 ± 84 Hz for testosterone females; Kruskal-Wallis Test; n = 6, p = 0.0374). An increase of higher frequencies due to increased sound pressure levels could explain this finding. Total production of soft song during the last three days before female robins were sacrificed was significantly higher in testosterone-treated females compared to the time-matched controls (mean ± SEM: 0.008 ± 0.006 min for controls and 146.8 ± 53.3 min for testosterone females; Kruskal-Wallis Test; n = 6, p = 0.0037). Occurrence of first loud song notes of testosterone-treated robins (Figures 1C and Additional file 1: Figure S1) were used as indicators for their ability to produce sound pressures that we observed in crystallized songs of our testosterone-treated female canaries.

Testosterone-induced song development is a well-described behavior of female canaries [24,53]. While our control female canaries did not sing at all, songs of testosterone-treated birds were highly structured (mean syllable repertoire: 10.3 syllables; mean tour length: 1.11 sec) with three out of six birds displaying fast frequency modulated syllables [24] in 15.94% of all tours.

### Morphological changes in the song control system of female robins

**Figure 2:**
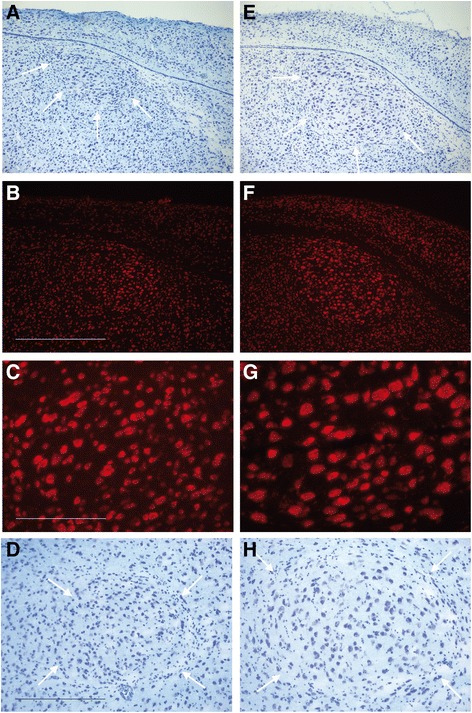
**Testosterone stimulated the growth of song control nuclei in female European robins.** Photomicrographs are shown for the HVC **(A-C, E-G)** and the RA **(D, H)** after staining with 0.1 % thionin (Nissl staining: **A**, **D**, **E**, **H**) and an anti-NeuN antibody **(B, C, F, G)**, respectively. Sagittal forebrain cryostat sections were obtained from control (left panels) and testosterone (right panels) female robins. Arrows indicate the border of a song control nucleus; scale bars represent 100 μm **(C** for **C** and **G)** and 500 μm **(B** for **B** and **F**; **D** for **D** and **H)**, respectively. Note that the testosterone treatment increased the volume **(A, B vs** **E, F; D vs** **H)**, neuron size and spacing **(C vs** **G)** of a song control nucleus.

For testosterone-induced effects on the adult female robins’ song control system, we looked at the neuroanatomy of the song control system and the size of the syrinx, the vocal organ. As expected from findings in adult female canaries [19,50-54], testosterone increased the size of song control nuclei in adult female robins (Figure 2). The volumes of HVC and RA as determined from Nissl-stained sections were found to have tripled after two weeks of testosterone treatment (mean ± SEM: 0.081 ± 0.012 mm^3^ for control-HVC and 0.271 ± 0.043 mm^3^ for testosterone-HVC; 0.079 ± 0.012 mm^3^ for control-RA and 0.26 ± 0.022 mm^3^ for testosterone-RA) (t-test: n = 4, t = 4.7337, p = 0.0031 for HVC and p = 0.0039 for RA).

**Figure 3:**
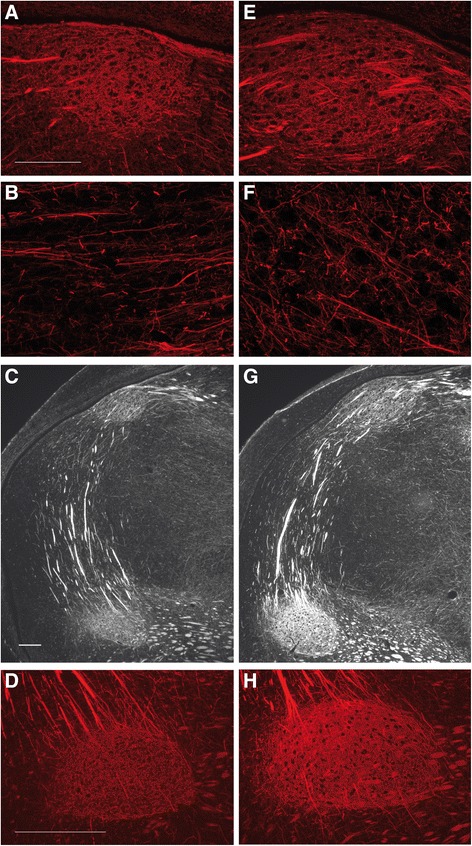
**Testosterone stimulated the expression of neurofilament in the song control nuclei of female European robins.** Photomicrographs are shown for the HVC **(A, B, E, F)**, the RA **(D, H)** and for the caudal forebrain **(C, G)** after anti-neurofilament immunofluorescence staining. Scale bars represent 100 μm **(A** for **A** and **E**; **C** for **C** and **G)** and 250 μm **(D, H)**, respectively. Sagittal cryostat sections were obtained from control (left panels) and testosterone (right panels) female robins. Please note an expansion of circumcellular, neurofilament-immunopositive structures after testosterone treatment.

Reduced density of NeuN-immunopositive cells after testosterone treatment (Figure 2: C vs G) suggested testosterone-induced neuronal spacing in HVC. In line with this observation we found spacing of nuclear signals in HVC to increase on average by a factor of 1.3 after two weeks of testosterone treatment (t-test; n = 4, t = 2.853, p = 0.0291) as determined by a counterstain with 4′,6-diamidino-2-phenylindole-dihydrochloride (DAPI) (Additional file 2: Figure S2). Circumcellular structures in HVC and RA were immunopositive for NEFM (neurofilament, medium polypeptide; Figure 3), which is a marker gene for HVC [63] that was found to be up-regulated by testosterone in our transcriptome study (Additional file 3: Table S1 and Additional file 4: Table S2). Since the testosterone treatment resulted in an enlargement of HVC volume but did not reduce the distribution of the anti-NEFM signal (Figure 3A,B vs E, F) HVC neuropil seemed to extend in response to the hormone. In register with this observation we determined the percentage of the NEFM-immunopositive area in HVC of control and testosterone birds to be identical after two weeks of testosterone treatment (mean ± SEM: 57.1 ± 1.6 % for control HVC and 57.9 ± 2.9 % for testosterone-HVC; t-test on the difference: n = 4, t = 0.2397, p = 0.8186). Strong anti-NEFM immunostaining of the RA after testosterone treatment (Figure 3C, D vs G, H) went together with testosterone-induced neuron projection morphogenesis in HVC (see below), but did not necessarily indicate enhanced RA innervations by HVC projection neurons.

In agreement with the effect of testosterone on the vocal organ in other songbird species [64-67], we detected syrinx growth in the adult female robins after testosterone treatment. Syrinx of control and testosterone females exhibited significant differences in both wet weight (mean ± SEM: 6.4 ± 0.3 mg for control and 9.6 ± 0.5 mg for testosterone females; p = 0.0062) and protein content as determined by a standard Bradford assay (mean ± SEM: 0.245 ± 0.018 mg for control and 0.335 ± 0.013 mg bovine serum albumin equivalents for testosterone females; p = 0.0106; Kruskal-Wallis Test, n = 6, p = 0.025 after Bonferroni correction).

### Testosterone effects on female HVC transcriptome

Transcriptome analysis of the female robin HVC revealed that the expression levels of 219 genes were increased after testosterone treatment by a factor of at least 1.6, while 281 genes were down-regulated by the same factor (Additional file 3: Table S1A). In the HVC of female canaries, the expression levels of 877 genes were up-regulated and decreased for 2127 genes after testosterone-treatment when applying the same criterion. In female robins, the expression of 50 % of the up- and 66 % of the down-regulated genes was concordantly changed by testosterone in female canaries as well (Additional file 5: Figure S3A). 

For the bioinformatic analyses of the transcriptome results we used information available for human orthologous genes. In the following, we will first describe testosterone-induced changes in cellular and biological processes in the HVC of adult female robins. Subsequently, we will compare the biological processes in the HVC that were affected by testosterone between female robins and canaries, focusing on processes that were enriched in the up- and down-regulated genes as determined by GeneRanker (Genomatix).

#### Effects in robins

Most of the genes that were strongly (> 1.8-fold) affected by testosterone showed an up-regulation in the HVC of female robins while down-regulated genes exhibited moderate expression changes (>1.6-fold; Additional file 3: Table S1B). We consistently identified processes related to cell locomotion and adhesion, neuron projection morphogenesis and axonal guidance, as well as blood vessel development to be most significantly overrepresented in the up-regulated genes (g:profiler; DAVID; GePS; IPA). Further, the largest network of up-regulated genes that we discovered by pathway analysis (GePS) (Additional file 6: file Figure S4) included well-known angiogenic growth factors like VEGFA (vascular endothelial growth factor A) and PGF (placental growth factor), as well as the VEGFA receptor KDR (VEGF receptor 2; [68]) and co-receptor NRP1 (neuropilin 1; [69]). Further, for most of the other genes that participated in this network, roles in blood vessel development have been reported. In addition, signaling processes involving the up-regulated ligands TNFSF13B (tumor necrosis factor (ligand) superfamily, member 13b), WNT7B (wingless-type MMTV integration site 7B) and HEG1 (heart of glass homolog 1) as well as the receptor MET (for hepatocyte growth factor) might have mediated pro-angiogenic testosterone effects [70-76]. Putative negative regulators of angiogenesis that were stimulated by testosterone were CXCL14 (chemokine ligand 14; [77]) and TNFRSF1 (tumor necrosis factor receptor superfamily, member 1A), which may have mediated the activity of up-regulated angiostatic extracellular matrix protein THBS1 (thrombospondin 1; [78,79].

In an additional functional transcriptome analysis we considered gene information obtained from PubMed and GenCards and limited the analysis to genes that showed at least two-fold changes in expression; as a consequence mainly up-regulated genes were included in this analysis. A majority (46 %) of the strongly responding genes affected cellular communication through processes like signal transduction, cell-cell/ECM interaction and growth factor activity (Additional file 4: Table S2).

#### Comparison of effects in robins and canaries

As expected from the diverging numbers of testosterone-regulated genes in the two bird species, we obtained unequal numbers of affected GO-terms (Additional file 7: Table S3; up-regulated: 285 for robin, 479 for canary; down-regulated: 150 for robin, 271 for canary). Most significantly overrepresented common GO-terms (up-regulated: 149; down-regulated: 56) in the group of up-regulated genes was blood vessel development (GO:0001568; p = 8.58E-10) and in the group of down-regulated genes was cell projection organization (GO:0030030; p = 1.2E-07). Angiogenesis and recruitment of newborn neurons are processes known to occur in the adult female canary HVC in response to testosterone and are considered to be closely linked [56]. Our transcriptome study of the HVC from testosterone-dependent female canaries revealed that neurogenesis (GO:0022008; p = 2.65E-08) was much more likely to be overrepresented in the up-regulated genes than angiogenesis (GO:0001525; p = 3.64E-04). In contrast, up-regulated genes in the HVC of our testosterone-treated adult female robins were more likely associated with blood vessel development (see above) and angiogenesis (p = 2.98E-05) than with neurogenesis (p = 8.78E-04) (Additional file 7: Table S3).

**Figure 4:**
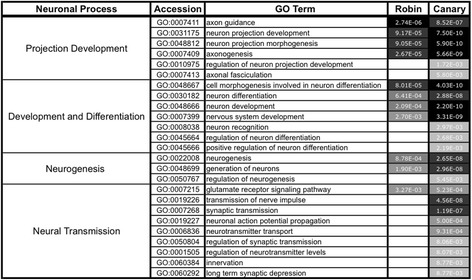
**Neuronal processes enriched in genes that were up-regulated by testosterone in the HVC of female European robins and canaries.** Probabilities with which GO-terms for neuronal processes were enriched in up-regulated genes were determined by GeneRanker (Genomatix) and ordered for robins within each neuronal process. Note that probabilities cannot be compared between species because of unequal total numbers of affected genes for each GO-term. Grey values indicate the probability of a biological process to be affected within a species. For complete lists of biological processes and explicitly neuronal processes enriched in down-regulated genes see Additional file 7: Table S3.

Regarding explicitly neuronal processes in the HVC of female robins, we found that testosterone induced the expression of genes that were most likely associated with the development of neuronal projections, the development and differentiation of neurons and with neurogenesis, although at a slightly lower probability (Figure 4). In female canaries, the same neuronal processes were affected by testosterone at relatively comparable probabilities. In contrast, synaptic transmission was enhanced by testosterone at a relative high probability only in the adult female HVC of canaries (Figure 4).

### Blood vessel development in the HVC of female robins

**Figure 5:**
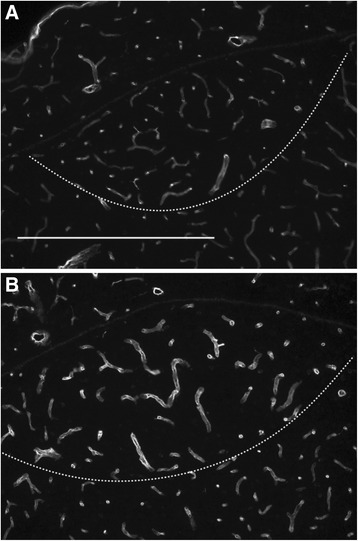
**Testosterone stimulated the growth of the vascular bed in the HVC of female European robins.** Photomicrographs of the HVC were prepared from control **(A)** and testosterone-treated **(B)** female robins and stained with an anti-laminin antibody. The ventral border of HVC was detected by a DAPI (4′,6-diamidino-2-phenylindole-dihydrochlorid) counterstain (data not shown) and is indicated by a dashed line; scale represents 500 μm.

Since transcriptome analysis revealed blood vessel development to be one of the most prominent effects of testosterone on the HVC of female robins, we investigated changes in the vascular bed in further detail. Using anti-laminin immunostaining of HVC endothelial cells [57] we observed a testosterone-induced enlargement of microcapillaries in the HVC of female robins (Figure 5). The average luminal diameter was increased by about 50 % after two weeks of testosterone treatment (mean ± SEM: 5.06 ± 0.05 μm in controls and 7.51 ± 0.36 μm in testosterone females; Kruskal-Wallis Test, n = 4 for controls and 6 for testosterone females, p = 0.0143).

### Expression and putative roles of BDNF in the HVC

**Figure 6:**
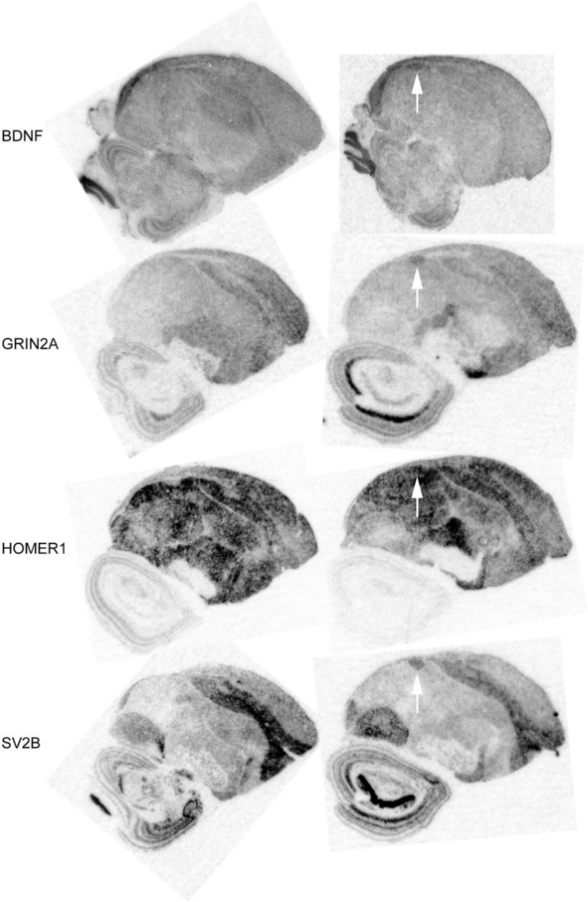
**Testosterone stimulated the expression of BDNF and its potential target genes in the HVC of female European robins.** Sagittal sections of control (left panels) and testosterone-treated (right panels) female robins were hybridized with radioactive antisense-probes for BDNF (brain-derived neurotrophic factor), and potential target genes of BDNF including the ionotropic glutamate receptor GRIN2A, HOMER1 that binds to metabotropic glutamate receptors and SV2B (synaptic vesicle glycoprotein 2b), respectively. Hybridized sections were exposed to an autoradiography film and the resulting autoradiographic images were scanned for documentation. For both groups images were representative for three (BDNF probe) and two birds (other probes), respectively. White arrows point to HVC.

Expression levels of BDNF as well as CADPS2 (Ca^2+^-dependent activator protein for secretion 2) that enhance activity-dependent neuronal BDNF release [80] were increased in adult female robin HVC after testosterone treatment (Additional file 3: Table S1; Figure 6). Bibliographic analysis (BiblioSphere, Genomatix) of gene interactions in the group of up-regulated genes revealed BDNF to be one of the four major interactome hubs beside VEGFA, FN1 (fibronectin 1) and HMGCR (3-hydroxy-3-methylglutaryl-CoA reductase). When we compared the testosterone-dependent transcriptome of the HVC between female robins and canaries, an up-regulation of BDNF expression turned out to be a common mechanism. Furthermore, the same response to testosterone was reported for the HVC of male Gambel’s white-crowned sparrows (Zonotrichia leucophrys gambelii) [81] (Additional file 5: Figure S3B). In addition to BDNF, three neuropeptides (chromogranin B, CHGB; neuropeptide Y, NPY; urotensin 2 domain containing, UTS2D) and a neuropeptide-regulating protein (corticotropin releasing hormone binding protein, CRHBP) seem to be conserved mediators of testosterone effects on the HVC in both sexes.

Previous studies of the role of testosterone-induced BDNF in the canary HVC focused on the recruitment and survival of newborn neurons [57,58]. To elucidate putative functions of BDNF as a mediator of testosterone effects on the HVC of female robins, we performed bioinformatic analyses (using GePS and IPA) of potential BDNF target genes that we found to be regulated in our transcriptome experiments (Additional file 8: Table S4). The results consistently revealed glutamate signaling (GePS: p = 10^−4^) or neurotransmission (IPA: p = 10^−6^), cell differentiation (GePS: p = 10^−3^; IPA: p = 10^−4^) and fibroblast/connective tissue cell proliferation (GePS: p = 10^−4^; IPA: p = 10^−3^) to be affected by potential BDNF genes in the HVC of testosterone-treated female robins. We obtained a comparable picture when focusing on potential BDNF target genes that were regulated in the HVC of female canaries. However, we found the processes learning or memory (GePS: p = 10^−8^) and regulation of apoptotic process/cell death (p = 10^−5^) to be affected by testosterone only in the HVC of female canaries and not in robins. Glutamatergic neurotransmission of female HVC cells might have been mediated by BDNF enhancing the expression of the ionotropic N-methyl-D-aspartate glutamate receptor 2A (GRIN2A), HOMER1 (homer homolog 1 (Drosophila)) that interacts with metabotropic glutamate receptors [82] and SV2B (synaptic vesicle glycoprotein 2 b) that plays a role in glutamate release [83] in both female robins and canaries. We performed in situ hybridization experiments to examine our transcriptome results that showed stimulatory effects of testosterone on the expression of these genes in the HVC of female robins. Outcomes of all hybridization experiments turned out to be consistent with the transcriptome study (Figure 6). However, sample numbers were low, so that additional experiments would be necessary to confirm testosterone induced expression changes in the female robin HVC as detected by the microarray analysis.

## Discussion

The singing activity of female European robins correlates with a naturally increased plasma testosterone level during the winter season [40]. In relation, song can be stimulated in female robins when endogenous plasma testosterone titers are low in spring [61] or in fall (this study) through a testosterone treatment. In the female canary, which has been the classical avian model for testosterone-induced song development, there is no evidence of a correlation between the plasma testosterone concentration and spontaneous female singing behavior. If anything, domesticated female canaries that sing spontaneously after the breeding season have lower plasma testosterone titers than breeding females [23]. Thus, use of female robins during the short day season is an alternative and novel approach to investigate physiological relevant testosterone effects on the song and song control system.

In female European robins captured in the fall, we observed an increase in soft song production within just a few days after the onset of testosterone administration while the time until occurrence of the first signs of full song varied considerably between birds. Different from robins, female canaries develop song under the influence of testosterone by progressively changing spectral features and temporal organization of notes [24,52,84] leading finally to the production of male-like song as in the case of the canaries of our study. Despite these differences in the effect of testosterone on singing behavior, we found European robins and canaries to share many neuroanatomical and gene expression changes in the song control system in response to experimental increases in testosterone levels. This congruence was all the more remarkable because in female robins we could not control for differences in features of the life history such as age and experience with the establishment of a winter territory.

An increase in the volume of the song control nuclei is one of the best-studied morphological effects of testosterone in female birds [19,85] and occurs in the HVC and RA of females from both species. In light of testosterone effects on the HVC such as neuronal soma enlargement and retention of dense neurofilament immunostaining of less densely packed neurons, one would predict the oxygen and energy demands of the HVC to be enhanced. Consistent with this expectation, the expansion of the vascular bed in the HVC is a prominent early effect of a testosterone treatment in canaries [51,56,57] and also occurred in female robins as shown in our transcriptome analysis and confirmed by anti-laminin immunostaining. Since angiogenesis requires the coordinated action of several cell types including neurons at the neurovascular interface [86], the differential regulation of some pleiotropic factors by testosterone could have reflected the complexity of the process of neurovascular congruence [86,87]. On a related note, TNFSF13B, the growth factor that showed the strongest up-regulation in the HVC of female robins in response to testosterone was described to be expressed by parenchymatic and perivascular astrocytes and assumed to play a role in the development of a central humoral immune response [88]. Later TNFSF13B was identified to inhibit neuronal outgrowth as well [89].

Patterns of gene expression in the same brain region in songbirds can show large differences even among closely related species [90] and comparable physiological effects of a testosterone treatment can correlate with divergent changes in the gene expression in the medial amygdala and the hypothalamus of male versus female juncos [91]. Nevertheless, a comparison of testosterone-stimulated HVC transcriptomes of female robins, female canaries (this study for both) and male white-crowned sparrows [81] suggested that BDNF and potential BDNF target genes are conserved mediators of testosterone actions on the HVC of songbirds. Since BDNF expression is up-regulated during singing in the HVC of both male canaries [92] and zebra finches [93], the observed effects of testosterone on BDNF expression in the HVC of female robins could have been indirect, for example resulting from hormonal actions on genes that are responsible for enhanced song production, instead of being the result of a direct control of the BDNF gene expression by the activation of sex hormone receptors.

Roles of BDNF in the song control system were reported in the context of neurogenesis and anterograde trophic support by previously published studies. In the adult female canary endothelial BDNF promotes neuronal differentiation and out-migration of ventricular zone cells [57] as well as the recruitment and survival of newborn cells in HVC [50,56,59]. In male Gambel’s white-crowned sparrows, the infusion of BDNF protein into RA increases cell soma size and decreases neuronal density [94]. In contrast to neurogenesis in female canaries, new neuron generation and recruitment in HVC turned out to be poorly stimulated by testosterone in female robins. While our pathway analysis of potential BDNF target genes that were regulated in the HVC supported a function of BDNF in the survival of newborn neurons in female canaries, similar processes were not detected in female robins using this subset of genes. However, in the female HVC of both robins and canaries locally acting BDNF most likely affected glutamatergic neurotransmission. By stimulating the expression of GRIN2A [95], a subunit of the NMDA glutamate receptor subtype, and HOMER1, a scaffold protein at the postsynaptic density that binds metabotropic glutamate receptors and modulates Ca^2+^ signaling [96], BDNF might have affected synaptic plasticity in HVC. An activity of BDNF that is different from recruitment and survival of newborn neurons that affects song is very likely to occur in the HVC of testosterone treated female canaries since BDNF-dependent singing of these birds precedes neuron recruitment [58]. 

Three potential target genes of BDNF that showed an increased expression level in the HVC of both female robins and canaries following a testosterone treatment are more strongly expressed in the HVC of testosterone-treated male Gambel’s white-crowned sparrows [81] as well (Additional file 5: Figure S3B): HOMER1, SV2B and NPY. Thus, the increased expression of these genes seems to be a common response of HVC to testosterone that might be mediated by BDNF.

In females of many bird species, selection seems to have favored loss of song behavior [14]. In at least two families of European passerine birds, the Muscicapidae (i.a. the European robin) and the Fringillidae/Carduelidae (i.a. the canary) female song appears to be an ancestral character [13]. In our transcriptome studies we found several biological processes including blood vessel development/angiogenesis and neuronal projection development along with increased expression of diffusible mediators like BDNF to be common testosterone effects in the HVC of female robins and canaries. These changes in gene expression are in agreement with the stimulatory testosterone effect on singing activity and might be part of an ancestral response of the song control system to testosterone. However, there were also differences in the patterns of gene expression of the female HVC following testosterone treatment including enhanced neurotransmission and potential BDNF effects on learning or memory particularly in canaries, and a relatively low incidence of a regulation of gene networks related to neurogenesis in robins. Whether these differences in the gene expression pattern are related to species-specific effects of testosterone on song development needs further investigation.

Divergent testosterone treatment regimes in our experiments could have caused species differences in the state of song development on the day of sacrifice and might have contributed to differences in the HVC transcriptome between female robins and canaries. However, it is questionable whether a relatively weak manifestation of neurogenesis in the HVC of female robins compared to canaries was a consequence of shorter periods of testosterone treatment in most female robins. In male canaries it takes about a week for newborn neurons to become sedentary in HVC [97]. For comparison, the shortest survival time of female robins after testosterone implantation was seven days in our experiments.

Strongly increased female song in both species following testosterone treatment and increased neurotransmission in the HVC of female canaries but not in robins may indicate species-specific regulatory mechanisms of song. In female robins of our experiments, testosterone stimulated the production of a constant flow of low amplitude notes as quiet as soft song of males signaling aggressive intent in other bird species [98]. In contrast, female canaries that are treated with testosterone utter high amplitude male-like songs that are characterized by much higher syllable repetition rates and longer repetitions of identical syllables (tours) [24,52,84] than any European robin song [45,99-101] (see also Additional file 1: Figure S1). Since HVC activity codes for the temporal order of song (for a review see [102]), the requirements for synaptic activity in HVC to produce testosterone-induced female song may be different between the two species. Strongly increased neurotransmission may be an essential testosterone effect to induce the temporal features of full song in female canaries, but dispensable for testosterone-stimulated song in female European robins. This observation justifies the assumption that testosterone-responsive mechanisms, which regulate song behavior in a bird species that was selectively bred for the elongation of tours [103], may not be involved in the stimulation of particular testosterone-responsive spontaneous song behaviors of wild bird species.

## Conclusions

We identified neuron growth and spacing, angiogenesis and neuron projection morphogenesis to be testosterone-induced effects in the HVC of female robins. Such cellular mechanisms of testosterone-induced HVC plasticity have already been described for the female canary, the model system for seasonal, sex hormone-dependent changes in bird song. While our transcriptome analysis of the female canary HVC revealed enhanced neurogenesis and synaptic transmission to be major neuronal responses to testosterone, the contribution of these processes to testosterone-induced HVC plasticity in robins was less prominent. Following testosterone treatment, females from both species showed an increased transcription level of BDNF that is believed to play a role in the recruitment and survival of newborn HVC cells. We confirmed these BDNF functions in the canary HVC by pathway analysis of potential BDNF target genes that were up-regulated after testosterone treatment. In contrast, testosterone effects on glutamatergic neurotransmission that might have been mediated by BDNF occurred in the HVC of both female robins and canaries.

## Methods

### Ethics statement

Animal handling was carried out in accordance with the European Communities Council Directive 2010/63 EU and legislation of the state of Upper Bavaria. Ethical approval of the experimental research on birds was received from the Upper Bavaria Commission of 4 April 2011 in accordance with the advisory procedure referred to in Article 15 of the German Animal Welfare Act. The government of Upper Bavaria, “Sachgebiet 54 - Verbraucherschutz, Veterinärwesen, 80538 München” with the record number 55.2-1-54-2532-169-11 approved animal experiments.

### Animals and testosterone treatment

Free-ranging European robin (*Erithacus rubecula rubecula*) females were collected by using ground traps during fall migration. Female canaries (*Serinus canaria*) were taken from the breeding facility of the Max Planck Institute for Ornithology and were part of a separate experiment examining the sex specificity of testosterone effects on the HVC in songbirds (M.-C. Ko et al., manuscript in preparation). Birds were housed individually in soundproofed recording boxes and song was recorded continuously as described in a previous study [104]. Birds were provided with water and food ad libitum while the artificial light cycle was adjusted weekly to natural conditions for sedentary robins. The canaries were kept on constant short day conditions (light: dark = 9.5 : 14.5 h). Blood samples were collected from the wing vein within five minutes after initial bird capture for sex determination by PCR using the primers P2 and P8 for the CHD1 alleles [105], and to determine testosterone plasma levels via radioimmunoassay as described in a previous study [106]. After recording the vocal activity of the birds for at least 14 days to determine individual baseline of singing activity, females were assigned randomly to either the testosterone (subcutaneous pellet; 1.5 mg; Innovative Research of America, USA) or control groups (n = 6 for all groups and both species).

Plasma testosterone levels from robins of both experimental groups were similar on the day of hormone pellet implantation (mean ± SEM: 42 ± 9 pg/ml for control birds vs 43 ± 12 pg/ml for testosterone females; Kruskal-Wallis Test; n = 6; p = 0.7445). In contrast, on the day of brain sampling plasma testosterone levels were significantly increased in the testosterone group as compared to the control group (7509 ± 1474 pg/ml for testosterone birds vs 29 ± 5 pg/ml for controls; p = 0.0045). While testosterone levels of control female robins did not differ before and after treatment (p = 0.4982), they did show significant increase in the testosterone group (p = 0.018). Thus, the experimental plasma level of testosterone was on average at least 7.5-fold increased compared to female robins living in the wild during the winter season (about 300–1000 pg/ml [40]). However, in testosterone-treated female canaries the plasma testosterone levels on the day of sacrifice were increased (6960 ± 1500 pg/ml for testosterone birds vs 108 ± 67 pg/ml for controls) to an extent we observed in female robins.

### Song recordings

Birds songs were continuously recorded and the sonograms of female robins were inspected daily to detect high amplitude song. Song files were analyzed using the Sound Analysis Pro software (http://soundanalysispro.com/). Since female European robins display full song in the context of territory conflicts during the winter season [44], we did not expect full song to develop under the housing conditions of this study. However, the housing conditions allowed monitoring of any changes in vocal production when treated with testosterone and sacrificing the female robins at the onset of high-amplitude note development. Experimental robins were sacrificed one day after we detected the first high amplitude notes with a frequency above 15 kHz in a certain individual (on day 7, 8, 9, 12, 32 and 40, respectively). An individual of the control group was sacrificed on the same day to ensure a time-matched sampling of individuals from both groups. The above time point of sampling was chosen first, to minimize the period of isolation for these wild-caught birds, and second, because the conditions and time female robins required for development of full song was not known. Canary brains were taken after 5 weeks of testosterone treatment when songs were crystallized.

### Microarray analysis

Birds were sacrificed by an overdose of isoflurane and brains were snap frozen on dry ice. Sagittal cryostat forebrain sections (30–40 μm) obtained from testosterone-treated females and time-matched controls were mounted on cold glass slides and stored at −80°C. To collect tissues, slides were thawed quickly and fixed in ethanol at room temperature for 5 seconds; the HVC was identified under a stereo-microscope after the addition of a drop of RNase-free PBS due to its location, degree of myelination and cell sizes. HVC samples were dissected manually with titanium forceps and transferred into QIAzol (Qiagen). RNA isolation was performed on the Qiacube according the manufacturer’s protocol for the Qiagen RNeasy Micro Kit (# 74004) pursuing the optional DNA digest step and RNA quality was assessed by using the Agilent Model 2100 Bioanalyzer (Agilent Technologies, Palo Alto, CA). RNA concentrations were assessed using a Nanodrop 1000 spectrometer (Thermo Fisher Scientific, Wilmington USA) and 100 ng total RNA was processed for hybridization on the microarray using the Ambion WT Expression Kit (# 4411974) and the Affymetrix WT Terminal Labeling and Controls Kit ( # 901524). The resulting cDNA was hybridized to the Custom Zebra finch Affymetrix Gene Chip® MPIO-ZF1s520811 Exon Array for 16 hours at 45°C and 60 rpm in the GeneChip Hybridization Oven 640. Samples from each bird were hybridized individually to separate arrays (n = 6 for canaries of both groups and testosterone-treated robins, and n = 5 for control robins because the RNA extraction of the 9d control bird failed). Arrays were washed, stained and scanned using the Affymetrix GeneChip Fluidics Station 450 and Affymetrix GeneChip scanner 3000 7G. CEL files were generated by the Affymetrix® GeneChip® Command Console® Software (AGCC) and the quality control for evaluating the success of individual hybridizations was assessed by the Affymetrix® Expression Console™ software.

Although the canary (Fringilidae) is more closely related to the zebra finch (Estrildidae) than the European robin (Muscicapidae) [107] and mean signal intensity was different between female European robins and canaries (mean ± SEM: 6.6 ± 0.02 for robins and 7.18 ± 0.01 for canaries; Kruskal-Wallis Test, p ≤0.001), equal numbers of probes were detected above and below the threshold of significance for detection in the two species (above: 212799 ± 1208 for robins and 213776 ± 416 for canaries; below: 19874 ± 1208 for robins and 18897 ± 416 for canaries; p >0.05). Since quality of array hybridization was similar in both species differences in GO term enrichment were not due to technical reasons.

Affymetrix CEL files were imported into the software ChipInspector 2 (Genomatix) for normalization, fold change calculation and statistical evaluation (http://www.genomatix.de/download/software/ChipInspector_Manual_V2) based on a single probe level [108]. Testosterone and control group were compared by probe wise division of the expression value of a testosterone-treated bird by expression values of all control birds. Mean expression data were analyzed using a minimum transcript coverage of 10-significant probes and a False Discovery Rate (FDR) set to zero. Genomic sequence differences were very unlikely to affect the detection of expression differences between species. Because on the array we were using any transcript was represented by several exon specific probe sets, and low probe coverage was sufficient for detection of transcript expression. Human orthologous gene IDs (g:Profiler, http://biit.cs.ut.ee/gprofiler/) of significantly affected genes were used for functional gene network analysis by publicly (g:Profiler; DAVID, http://david.abcc.ncifcrf.gov/) as well as commercially (GePS, BiblioSphere Pathway, Genomatix, Germany; IPA, Ingenuity Pathway Analysis, Ingenuity Systems, USA) available softwares [109,110]. In addition, we considered gene information obtained from PubMed (http://www.ncbi.nlm.nih.gov/pubmed) and GenCards (http://www.genecards.org/).

### Histological analyses

To support selected findings of the transcriptome analysis from the HVC of female robins we performed histological analyses on 14 μm forebrain sections that were obtained in parallel with the cryostat sections used for the microarray study. Thus, for each bird we produced several sets of sections that included thicker sections for the microarray analysis and singly mounted 14 *μm* sections that were submitted to histological staining procedures and the in situ hybridization experiments, respectively. For verification and quantification of observations we made in birds of the microarray study we used HVC sections of female robins that obtained implants for two weeks (n = 4). Sections were mounted on SuperFrost microscope slides and Nissl and nuclear DAPI (4′,6-diamidino-2-phenylindole-dihydrochlorid, Merck) stainings were performed following standard protocols. Approximate HVC volumes were calculated using Nissl-stained sections as the sum of the area sizes multiplied by the product of section interval and section thickness. To estimate cell spacing we determined the number of HVC cells in a defined area (1500 × 1500 pixel) of microscopic DAPI-images (32-fold magnification; 6 images per bird) with the help of ImageJ (http://rsb.info.nih.gov/ij/). For immunostaining of HVC endothelium we used a rabbit polyclonal anti-Laminin antibody (Sigma, L9393) as the primary antibody diluted 1 : 100 in 1.9 mM KH_2_PO_4_, 8.1 mM Na_2_HPO_4_x7H_2_O, 150 mM NaCl (pH 7.4) and 0.1 % Triton X-100 (all Sigma) that was supplemented with 10 % non-immune goat serum (Invitrogen). For immunostaining of HVC neurons primary antibodies were mouse monoclonal antibodies for NeuN (MAB377; 1:50) and Neurofilament (MAB5254; 1:200) (both Merck Millipore) diluted in 0.5 mM NaH_2_PO_4_ x 2H_2_O (Sigma), 15 mM Na_2_HPO_4_x7H_2_O, 250 mM NaCl (pH 7.4), 0.1 % Triton X-100 and 10 % non-immune goat serum. Secondary antibodies were goat IgGs conjugated with Alexa Fluor 488 (anti-rabbit) and 555 (anti-mouse) (both Invitrogen; 1:500), respectively. Fluorescent images were obtained with a LEICA DM 6000 B (Wetzlar, Germany) microscope. Luminal diameter of HVC microcapillaries was determined at 100-fold magnification (Leica HCX PL APO 100x/NA1.4 oil immersion objective) with the help of the LEICA Image Manager. Both transversal and longitudinal capillary profiles were considered. To estimate the distribution of fluorescent anti-NEFM signal in HVC microscopic images (40-fold magnification; 4 sections per bird) were obtained under identical conditions. With the help of ImageJ using default colour threshold settings we determined total area and area covered by anti-NEFM immunostaining.

In situ hybridizations were performed as described in a previous study [111]. RNA-probes specific to European robins were obtained by doing PCRs with adult male robin forebrain cDNA. Based on zebra finch sequence information PCR primers were designed for BDNF (NM_001048255.1; Fw: 5′-ATGACCATCCTTTTCTTTACTATG-3′; Rv: 5′- TCTTCCCCTTTTAATGGTCAATGTAC-3′) and potential BDNF target genes like glutamate receptor, ionotropic, N-methyl D-aspartate 2A (GRIN2A; ENSTGUT00000004978; Fw: 5′-TGCATCAGCCATGCTGGAAAA-3′; Rv: 5′-CATCACCCAGACAGAAGCACTA-3′), homer homolog 1 (Drosophila) (HOMER1; ENSTGUT00000003868; Fw: 5′-GCATGCTGTTACTGTGTCCTACT-3′; Rv: 5′-TGCTCATTCTGACTTTTCTCCA-3′) and synaptic vesicle glycoprotein 2B (SV2B; ENSTGUT00000008813; Fw: 5′-GCTGGCTGTGGTTTGGTTTAC-3′; Rv: 5′-GGCACCAAACTTGCAAAGCC-3′). The pCRII-TOPO vector (Life Technologies) was used to clone and sequence the RT-PCR products and to generate ^35^S-CTP-labeled anti-sense probes. Processed slides were exposed to Biomax XAR film.

### Availability of supporting data

Raw microarray data generated in this study have been deposited in the NCBI Gene Expression Omnibus (GEO). They are available for female European robins http://www.ncbi.nlm.nih.gov/geo/query/acc.cgi?acc = GSE59203 and female canaries http://www.ncbi.nlm.nih.gov/geo/query/acc.cgi?acc = GSE59310. All other supporting data were included as additional files.

## Additional files

Additional file 1: Figure S1. Small sections of soft song and full song notes from European robins. (A-E) Small sections from the continuous stream of notes of soft song (ss) and first high amplitude full song notes (fs) are presented for the remaining five females (for songs of the sixth female see Figure 1 in the main text). (F) Small portion of full song from a male European robin. Sound waves (amplitude (analog/digital arbitrary units) over time (ms)) are shown in the upper panels and sonograms are presented in the lower panels (frequency (Hz) over time (ms)). Additional file 2: Figure S2. Nuclear stain of the HVC was used for estimate the testosterone effect on cell density in the HVC of female European robins. Photomicrographs are shown for the HVC of control (A - D) and testosterone-treated (E - H) birds after staining with 4′,6-Diamidino-2-phenylindole dihydrochloride (DAPI) visualizing nuclear DNA. Arrows point at the epithelium of the collapsed lateral ventricle dorsal of HVC. The scale bar represents 500 μm. Additional file 3: Table S1. Genes that were differentially expressed in the HVC of female European robins after testosterone treatment. Genes were identified by microarray analysis with the Custom Zebra finch Affymetrix Gene Chip® MPIO-ZF1s520811 and were listed as the human orthologous genes. (A) Lists of genes that were at least 1.6-fold up- or down-regulated. (B) Number of genes that showed the indicated fold change. Additional file 4: Table S2. Genes that showed strong expression changes (≥2-fold) after testosterone treatment in the HVC of female European robins. Genes were assigned to functional categories according to information obtained from PubMed (http://www.ncbi.nlm.nih.gov/pubmed) and GenCards (http://www.genecards.org/). Additional file 5: Figure S3. Venn diagrams of genes differentially expressed in HVC after testosterone treatment. (A) Congruence of up- and down-regulated genes between female robins (red) and canaries (yellow). (B) Genes that were up-regulated in female European robins and canaries (both from this study), and in male white-crowned sparrows [81]. Symbols are given for genes that were up-regulated in the HVC of all three species and BDNF as well as potential BDNF target genes (Additional file 8: Table S4) are highlighted using bold letters. Additional file 6: Figure S4. Genes that showed expression changes after testosterone treatment and are expected to play a role for the blood vessel development in the HVC of female European robins. (A) The largest network of up-regulated genes that we discovered by GePS (Genomatix) analysis included two angiogenic ligands (VEGFA, PGF). Further, (B) an association with angiogenic processes has been reported for most of the other genes of this network. Additional file 7: Table S3. Enrichment of biological processes in genes that showed expression changes after testosterone treatment in the HVC of female European robins and canaries. Probabilities of biological processes enriched in (A, B) up- and (C, D) down-regulated genes expressed in the HVC of robins (A, C) and canaries (B, D), respectively, were determined by GeneRanker (Genomatix). For (E) explicitly neuronal GO-terms were selected from the list of genes that were down-regulated in the HVC of female robins genes and grouped as shown for up-regulated genes in Figure 4 of the main text. For comparison probabilities of the same processes are also shown for female canaries. Enrichment of neuron projection development, neuron development/differentiation and neurogenesis in both up- and down-regulated genes suggested that these processes were dynamically affected following the testosterone treatment. Additional file 8: Table S4. Potential BDNF target genes. (A) Potential BDNF target genes that were up- or down-regulated after testosterone treatment in the HVC of female robins. An asterisk indicates response to testosterone in the HVC of female canaries. (B) Additional potential BDNF target genes not affected by testosterone in the HVC of female robins HVC. For references that indicate BDNF-dependent changes in other species see the end of the document. As expected from the multiple cellular functions of BDNF the bioinformatic analysis (GePS; IPA) of all potential BDNF target genes revealed consistently cellular processes including neurotransmission and synaptic plasticity, regulation of cell death/apoptosis as well as cell growth, differentiation and proliferation.

## Abbreviations

BDNF: Brain-derived neurotrophic factor
CADPS2: Ca^2+^-dependent secretion activator 2
DAPI: 4′,6-diamidino-2-phenylindole
ECM: Extracellular matrix
GRIN: Glutamate receptor, ionotropic, N-methyl D-aspartate
Homer: Homer homolog (Drosophila)
HVC: High vocal center
KDR: Kinase insert domain receptor
KITLG: KIT ligand
l/mMAN: Lateral/medial magnocellular nucleus of the anterior nicopallium
MET: Met proto-oncogene
NEFM: Neurofilament, medium polypeptide
PGF: Placental growth factor
RA: Robust nucleus of the arcopallium
SV2B: Synaptic vesicle glycoprotein 2B
VEGF: Vascular endothelial growth factor
