# Supplementary figures and images for: Regulatory mechanisms of testosterone-stimulated song in the sensorimotor nucleus HVC of female songbirds
Source: BMC Neurosci. 2014 Dec 2;15:128. doi: 10.1186/s12868-014-0128-0 (PMC4261767; doi:10.1186/s12868-014-0128-0)

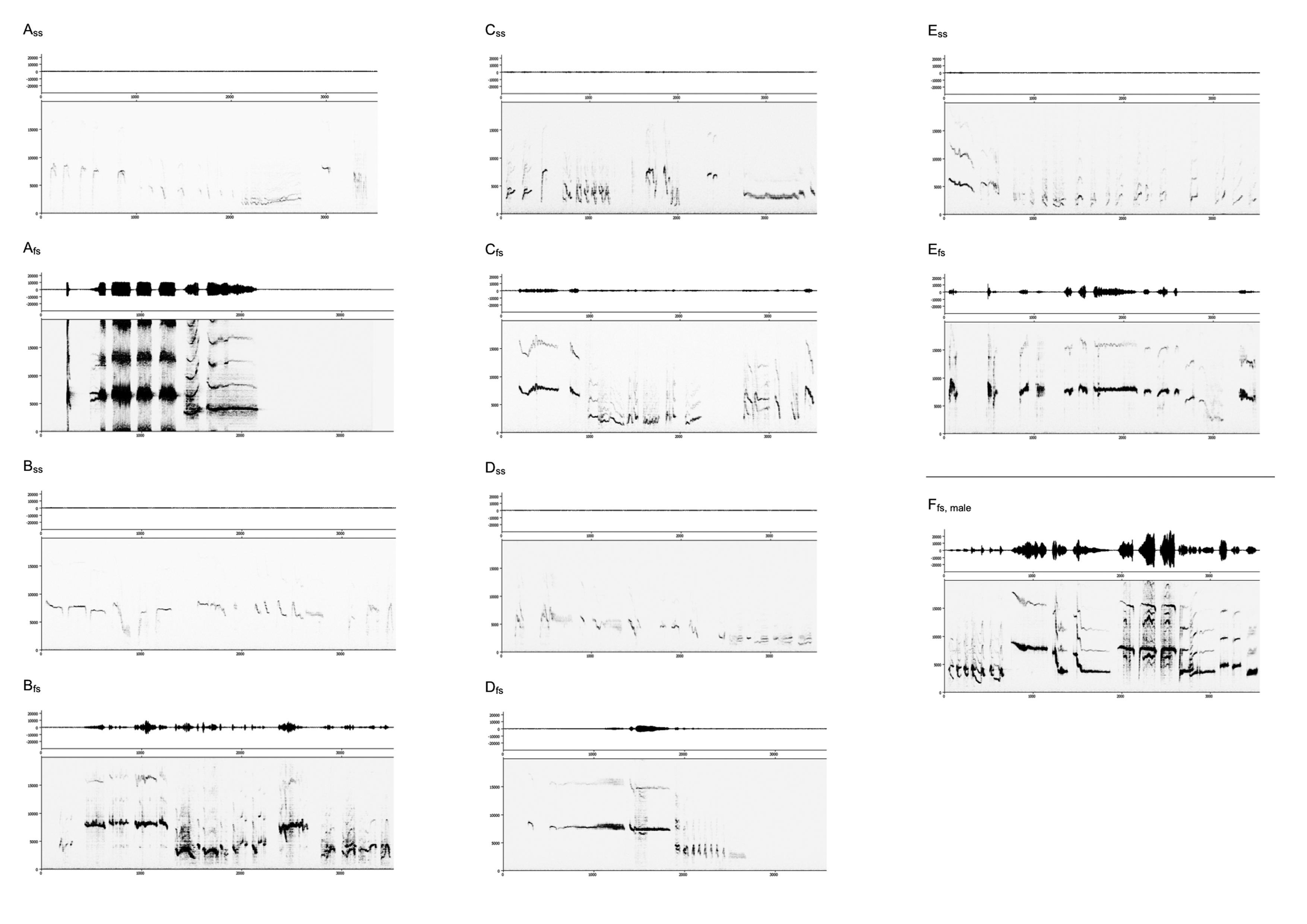

Supplement: Additional file 1: Figure S1. — Small sections of soft song and full song notes from European robins. (A-E) Small sections from the continuous stream of notes of soft song (ss) and first high amplitude full song notes (fs) are presented for the remaining five females (for songs of the sixth female see Figure 1 in the main text). (F) Small portion of full song from a male European robin. Sound waves (amplitude (analog/digital arbitrary units) over time (ms)) are shown in the upper panels and sonograms are presented in the lower panels (frequency (Hz) over time (ms)). [file 12868_2014_128_MOESM1_ESM.tiff]

Control

Testosterone

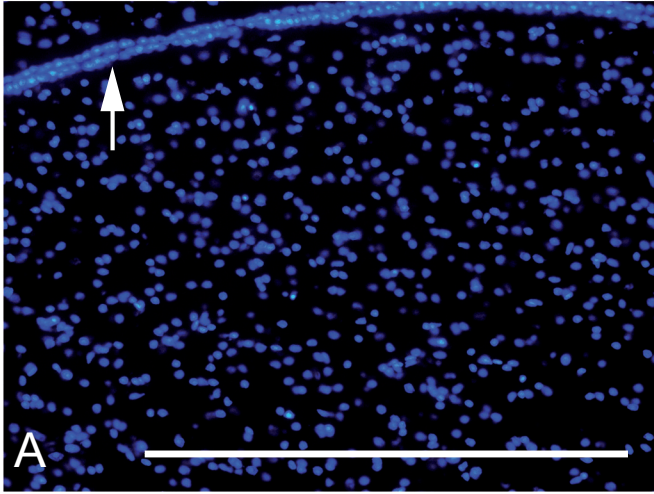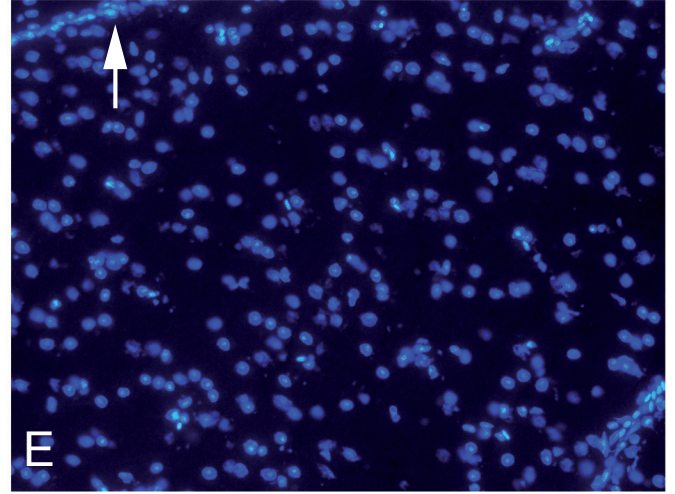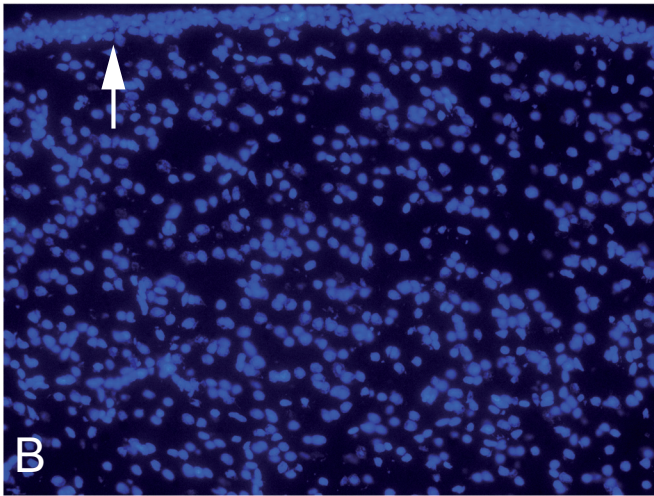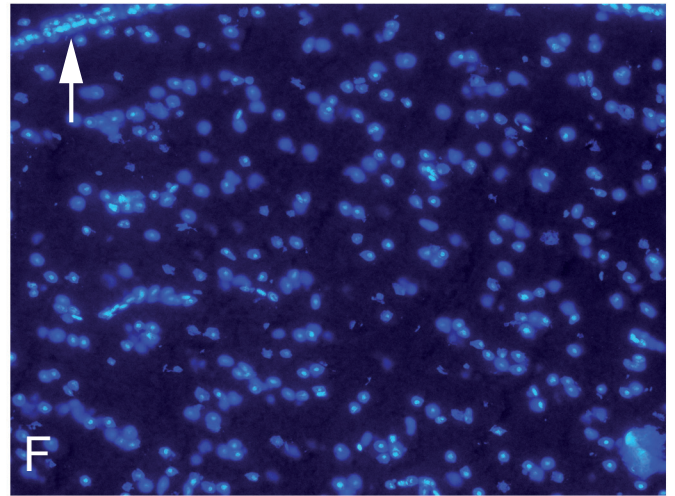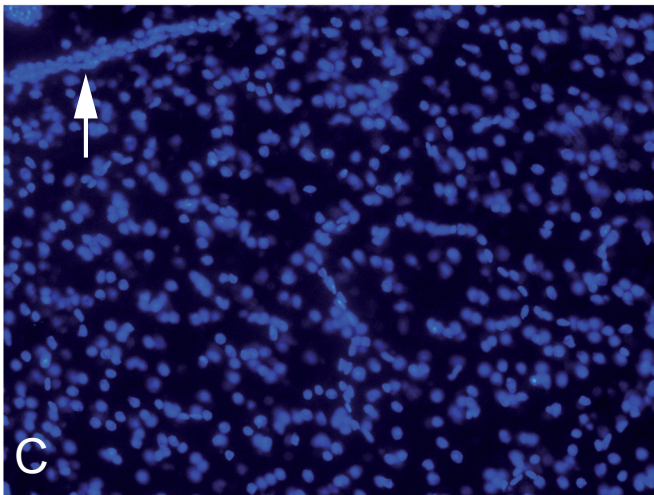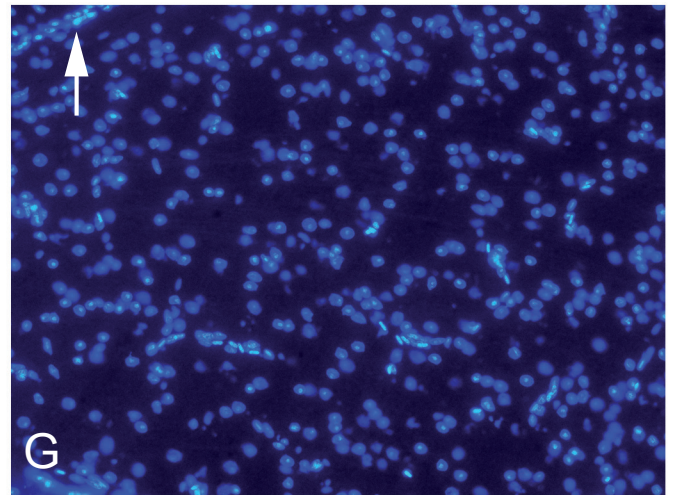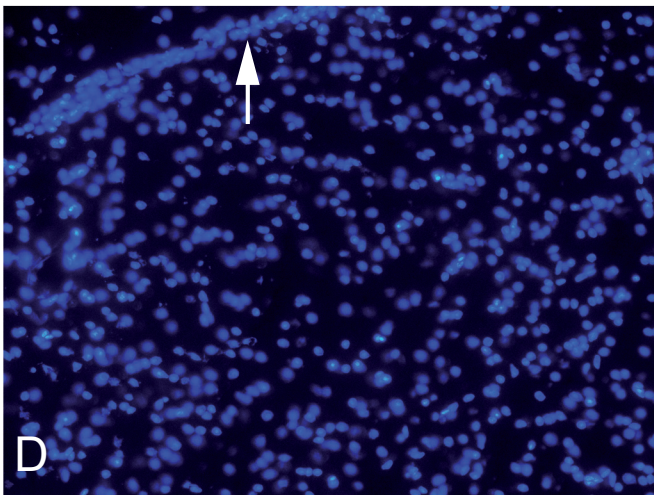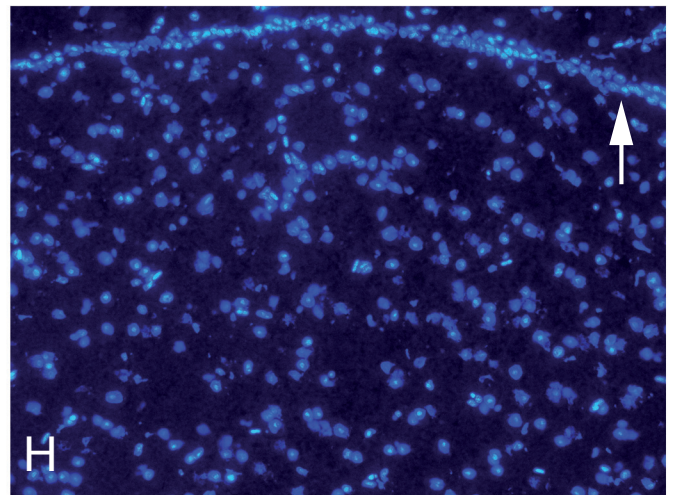

Supplement: Additional file 2: Figure S2. — Nuclear stain of the HVC was used for estimate the testosterone effect on cell density in the HVC of female European robins. Photomicrographs are shown for the HVC of control (A - D) and testosterone-treated (E - H) birds after staining with 4′,6-Diamidino-2-phenylindole dihydrochloride (DAPI) visualizing nuclear DNA. Arrows point at the epithelium of the collapsed lateral ventricle dorsal of HVC. The scale bar represents 500 μm. [file 12868_2014_128_MOESM2_ESM.pdf]

A

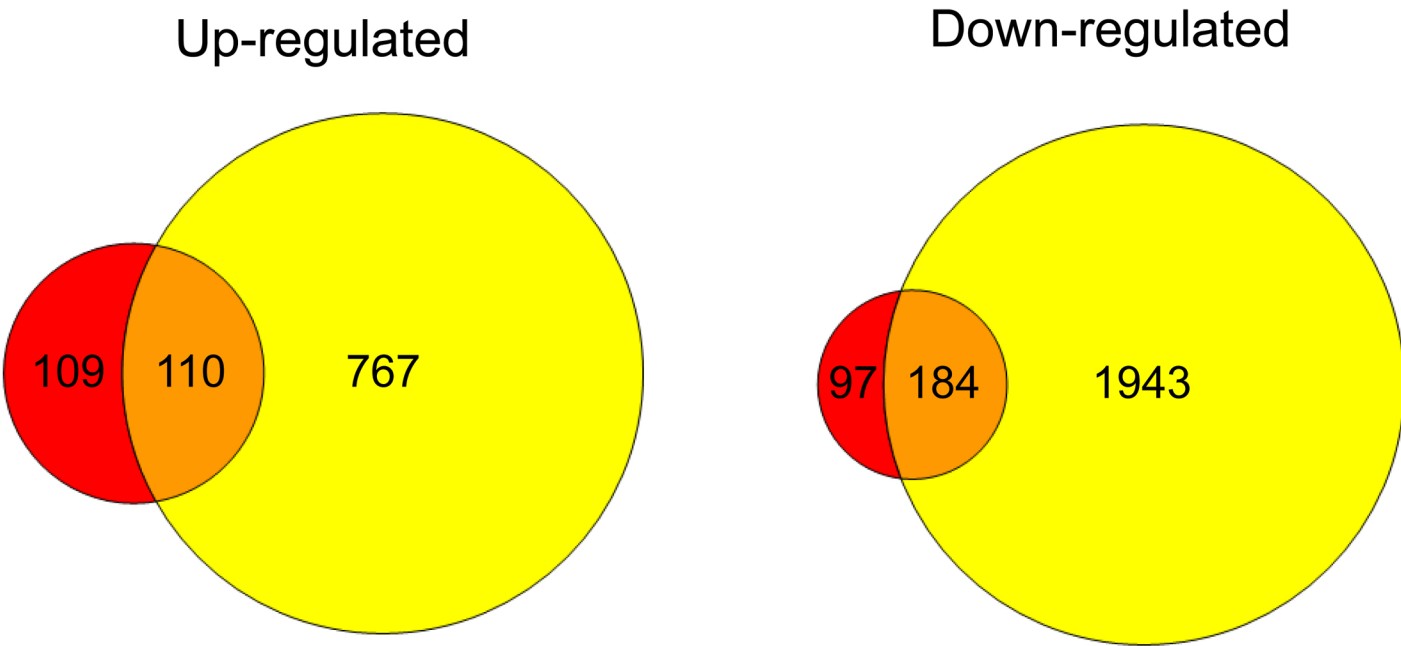

B

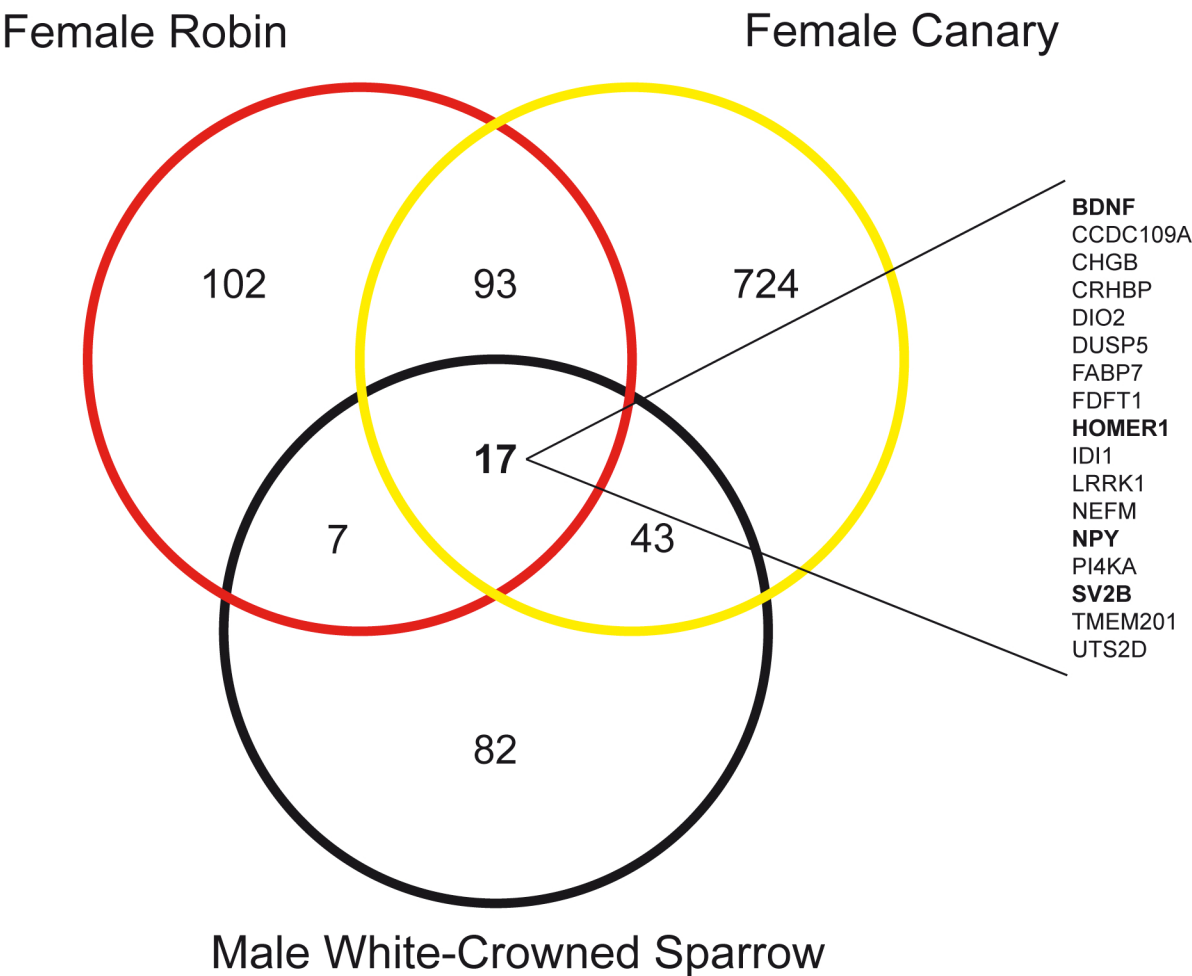

Supplement: Additional file 5: Figure S3. — Venn diagrams of genes differentially expressed in HVC after testosterone treatment. (A) Congruence of up- and down-regulated genes between female robins (red) and canaries (yellow). (B) Genes that were up-regulated in female European robins and canaries (both from this study), and in male white-crowned sparrows [81]. Symbols are given for genes that were up-regulated in the HVC of all three species and BDNF as well as potential BDNF target genes (Additional file 8: Table S4) are highlighted using bold letters. [file 12868_2014_128_MOESM5_ESM.pdf]
