# Supplementary material for: Regulatory mechanisms of testosterone-stimulated song in the sensorimotor nucleus HVC of female songbirds
Source: BMC Neurosci. 2014 Dec 2;15:128. doi: 10.1186/s12868-014-0128-0 (PMC4261767; doi:10.1186/s12868-014-0128-0)
Supplement: Additional file 3: Table S1. — Genes that were differentially expressed in the HVC of female European robins after testosterone treatment. Genes were identified by microarray analysis with the Custom Zebra finch Affymetrix Gene Chip® MPIO-ZF1s520811 and were listed as the human orthologous genes. (A) Lists of genes that were at least 1.6-fold up- or down-regulated. (B) Number of genes that showed the indicated fold change. [file 12868_2014_128_MOESM3_ESM.pdf]

A

Up-regulated		Down-regulated	
Symbol	Fold change	Symbol	Fold change
ABCB1	1.693490625	A2M	1.815038329
ABCC2	1.693490625	ABCA1	1.741101141
ACLY	1.658639092	ABCC8	1.905276006
ACSBG2	1.945309895	ABCC9	1.802500955
ACTN2	2.361985323	ACOX2	1.635804093
ADAM23	1.892115293	ADAMTS5	1.729074488
ADAMTS9	1.853176124	AKAP9	1.705269761
ADAMTS20	1.729074463	ALDH3A2	1.765405984
ADD3	1.647182035	ALS2CR8	1.602139763
ADSSL1	1.717130873	ANKDD1A	1.753211446
AFF3	1.777685362	ANKFN1	1.681792831
ALDH1A2	2.203810232	ANKRD5	1.802500955
AOAH	1.729074463	ANKS1B	1.658639114
AR	1.972465409	ANXA7	1.635804093
ARHGAP31	1.8276629	ARHGAP32	1.67017585
ARPP19	1.647182035	ARHGEF5	1.647182067
ASTN2	1.879045498	ASAH2	1.802500955
ATP2A3	2.099433367	ATP1A1	1.693490614
ATP5A1	1.647182035	ATF7IP	1.905276006
ATP6V1C1	2.234574276	ATP9A	1.658639114
BACE2	1.729074463	ATP10D	1.624504779
BDNF	2.234574276	B3GNTL1	1.705269761
BRS3	2.084931522	BAZ2B	1.693490614
C18orf1	1.8276629	BBX	1.647182067
C4A	1.753211443	BCAS3	1.647182067
CADPS2	1.945309895	BICC1	1.635804093
CAMK1D	1.705269784	BOK	1.647182067
CAR15	1.741101127	BRCA2	1.879045533
CASK	1.624504793	C2orf43	1.815038329
CCDC109A	1.613283518	C5orf4	1.729074488
CCDC134	1.693490625	C5orf41	1.765405984
CDH5	1.658639092	C6orf103	1.717130839
CHGB	1.815038311	C6orf170	1.705269761
CHST9	2.0139111	C7orf63	1.802500955

CNTNAP1	1.693490625	C14orf49	1.681792831
COL4A1	1.972465409	C16orf70	1.765405984
COL4A2	1.879045498	CABIN1	1.635804093
COL4A5	1.879045498	CAMK1G	1.729074488
COL6A1	2.566851795	CBFA2T2	1.67017585
COL6A3	1.866065983	CC2D2A	1.681792831
COL19A1	1.931872658	CD2AP	1.681792831
CRHBP	2.867910496	CDH9	1.624504779
CXCL14	1.693490625	CDH23	1.67017585
CXXC5	1.705269784	CDK19	1.635804093
CYB5A	1.635804117	CDKL1	1.753211446
DAPK1	1.670175839	CDON	1.741101141
DBC1	1.729074463	CELSR1	1.681792831
DCTN3	1.705269784	CEP110	1.658639114
DHCR7	1.705269784	CEP112	1.647182067
DHX29	1.705269784	CHD2	1.613283516
DHX35	1.658639092	CHD5	1.765405984
DIO2	2.281527432	CHD9	1.840375295
DNAJB5	2.329467173	CHL1	1.635804093
DOK7	1.945309895	CLCN6	1.729074488
DOS	1.765405993	CNNM1	1.635804093
DOT1L	1.658639092	CNTN3	1.624504779
DPP4	1.918528239	CNTNAP5	1.624504779
DPP10	1.647182035	COL4A4	1.67017585
DPT	2.02791896	COL12A1	1.790050109
DUSP5	2.313376368	COL27A1	1.753211446
DUSP6	1.8276629	CORIN	1.635804093
DUSP7	1.777685362	CRHR1	1.681792831
EDA2R	2.143546925	CSMD2	1.705269761
EMCN	2.02791896	CTR9	1.765405984
ESYT3	1.765405993	CUBN	1.777685342
F13A1	1.945309895	CYTH1	1.777685342
FABP7	1.918528239	DDX51	1.705269761
FADS2	3.138336392	DENND1A	1.840375295
FAM13A	1.853176124	DENND4A	1.613283516
FAM13C	1.777685362	DEPDC5	1.635804093
FAM126A	1.741101127	DMTF1	1.624504779

FANCA	2.099433367	DMXL2	1.945309866
FAT1	1.729074463	DNAH1	1.705269761
FDFT1	1.624504793	DNAH3	1.67017585
FGFR2	1.693490625	DNAH5	1.67017585
FKBP5	1.815038311	DNAH9	1.802500955
FN1	1.815038311	DNAH10	1.729074488
FOSL2	1.705269784	DNAH12	1.693490614
GFRA1	2.234574276	DOCK4	1.635804093
GGT5	1.741101127	DOCK10	1.635804093
GPC3	1.931872658	DOPEY2	1.705269761
GRIN2A	1.681792831	DSCAM	1.635804093
HECTD2	1.705269784	DST	1.658639114
HEG1	1.777685362	ECEL1	1.777685342
HERC2	2.694467154	EEF2K	1.717130839
HMGCR	1.681792831	EFEMP1	1.624504779
HMGCS1	1.741101127	EGFR	1.705269761
HOMER1	1.681792831	EIF4ENIF1	1.647182067
HSD17B12	1.931872658	EIF2AK4	1.753211446
HTR7	1.777685362	EML6	1.67017585
IARS	1.670175839	ENOX1	2.20381021
IDI1	1.802500925	EP300	1.613283516
IFRD1	1.729074463	EP400	1.658639114
IGF2	1.958840595	EPB41L2	1.777685342
IGSF9B	1.717130873	EPB41L3	1.717130839
IMPA1	1.670175839	EPB41L4B	1.681792831
INCENP	1.717130873	EPB41L5	1.705269761
INF2	1.753211443	ERBB4	1.602139763
INSIG1	1.741101127	EYA1	1.717130839
IPO11	1.905275996	EZH1	1.658639114
ITGA1	1.815038311	F3	1.765405984
ITPR3	1.658639092	FAM70B	1.815038329
JARID2	1.802500925	FAM184A	1.802500955
KCNK16	1.853176124	FAM196A	1.635804093
KDR	2.042024251	FARP2	1.729074488
KIAA1217	1.624504793	FAT3	2.219138907
KIAA1239	1.729074463	FBN2	1.802500955
KITLG	1.693490625	FCGBP	1.840375295

KLHL4	1.624504793	FNBP1L	1.931872642
KRT1	2.219138944	FOXP1	1.717130839
KTN1	2.514026749	FOXP2	1.681792831
LAMA2	1.658639092	FRAS1	1.67017585
LAMA4	1.765405993	FRMPD2	1.693490614
LMBRD1	3.095129987	FRMPD3	1.790050109
LMBRD2	1.658639092	GAB3	1.705269761
LRRK1	1.945309895	GABRB1	1.729074488
LTA4H	1.658639092	GATSL2	1.693490614
LY75	1.802500925	GHR	1.753211446
MACF1	2.02791896	GLI3	1.681792831
MAP4K4	1.705269784	GPLD1	1.658639114
MAPK6	2.188587403	GPR98	1.693490614
MAPK8IP1	1.741101127	GPR123	1.681792831
MAPK11	1.729074463	GRAMD1C	1.717130839
MAPK13	2.158456473	GTF2H4	1.693490614
MET	1.958840595	GUCY1B2	1.681792831
MMRN2	1.986184991	HDAC4	1.624504779
MPP1	3.073750363	HELZ	1.658639114
MPP6	1.705269784	HHIP	1.866065952
MYH9	1.681792831	HMCN2	1.753211446
MYO5B	1.693490625	HNF4A	1.753211446
MYO10	2.394957409	IFT140	1.658639114
NAA35	1.635804117	IGF1R	1.717130839
NEFM	2.084931439	INPP5B	1.635804093
NEK5	1.705269784	INTS2	1.67017585
NETO1	1.602139755	INTU	1.693490614
NID1	1.853176124	ITGA8	1.705269761
NPY	1.972465409	KCNH7	1.67017585
NRK	1.705269784	KDM2B	1.658639114
NRN1	1.717130873	KIAA0430	1.693490614
NRP1	1.705269784	KIAA0564	1.693490614
NSDHL	1.741101127	KIAA1109	1.681792831
NSUN2	1.624504793	KIAA1244	1.613283516
NUP62CL	1.635804117	KIAA1409	1.717130839
OBFC2A	1.658639092	KIF1B	1.624504779
ODZ1	2.462288827	KIT	1.717130839

OSBPL8	1.866065983	LAMA3	1.705269761
PDCD6	1.729074463	LAMA5	1.681792831
PDE4D	2.02791896	LGR5	1.717130839
PDE5A	2.0139111	LPHN2	1.658639114
PECAM1	1.753211443	LRBA	1.729074488
PGF	2.158456473	LRMP	1.717130839
PI4KA	2.928171392	LRP1B	1.67017585
PIK3C2A	2.584705661	LRP2	1.705269761
PIM3	1.658639092	LRRC7	2.549121297
PITPNC1	1.635804117	LRRCC1	1.67017585
PLEKHO2	1.705269784	LRRK2	1.681792831
PLIN2	1.693490625	LYPD1	1.613283516
PLK3	1.741101127	MAP7	1.905276006
POLQ	1.8276629	MC4R	1.972465435
PSMA3	1.635804117	MDN1	1.705269761
PTP4A3	1.879045498	MED12	1.753211446
PTPRB	1.765405993	MED13L	1.624504779
PYGB	1.790050142	MED15	1.840375295
RASGRP1	1.729074463	MFGE8	1.918528235
RDH10	1.853176124	MGAT5B	1.681792831
RGL1	1.802500925	MIER2	1.972465435
RGS9	1.840375301	MLL	1.693490614
RHBDF1	1.670175839	MLL3	1.613283516
RHOB	1.777685362	MOXD1	1.892115315
RRAD	1.945309895	MPPED1	1.647182067
RRP9	1.613283518	MRVI1	1.790050109
SCGN	1.892115293	MTA3	1.647182067
SDC1	1.8276629	MYCBP2	1.729074488
SEMA3C	1.777685362	MYH3	1.693490614
SIDT1	1.670175839	MYO1E	1.681792831
SLC6A17	1.945309868	MYOF	1.705269761
SLC30A5	1.717130873	NCKIPSD	1.602139763
SLC35F3	1.777685362	NCOA2	1.647182067
SLC40A1	1.945309895	NCOA6	1.840375295
SNAI2	2.0139111	NEDD4	1.67017585
SORCS3	1.741101127	NOS1	1.613283516
SPAG9	2.29739671	NOV	1.693490614

SPHKAP	1.658639092	NPR2	1.693490614
SPINK5	2.143546925	NTSR1	1.777685342
SPTAN1	2.143546925	OBSCN	1.705269761
SRD5A2	1.905275996	OSBPL5	1.635804093
SRPR	1.635804117	OTOG	1.717130839
SSBP3	2.394957409	OTOGL	2.013911087
ST6GAL2	1.8276629	PALLD	1.658639114
STARD13	1.790050142	PCDP1	1.693490614
STC2	1.931872658	PDE7B	1.624504779
STOML2	1.670175839	PER2	1.693490614
SUPV3L1	1.853176124	PEX5L	2.20381021
SV2B	2.29739671	PHIP	1.918528235
SYNM	1.790050142	PHKA2	1.693490614
TBX3	1.905275996	PITPNM2	1.693490614
THBS1	1.693490625	PKHD1	1.729074488
TMC3	1.931872658	PLCH2	1.717130839
TMEM201	2.219138944	PLEKHA7	1.647182067
TMSB4X	1.717130873	PLXNC1	1.705269761
TNFRSF1A	1.8276629	PLXND1	1.705269761
TNFSF13B	2.907945035	PMS1	1.705269761
TRHR	2.0139111	POLA1	1.741101141
TRPC4	1.802500925	POU2F1	1.624504779
TRPM2	1.717130873	PPFIA1	1.765405984
TRPM8	1.931872658	PPFIBP1	1.67017585
UBXN2A	1.931872658	PREPL	1.693490614
UNC13C	1.905275996	PRKAR1B	1.741101141
USP12	1.681792831	PRKDC	1.705269761
USP15	1.635804117	PRKG2	1.681792831
UTS2D	2.203810232	PTPN13	1.717130839
VCL	1.635804117	RAB32	1.717130839
VEGFA	1.802500925	RASD2	1.705269761
WDR36	1.8276629	RBL2	1.729074488
WDR41	1.705269784	REPS2	1.681792831
WFDC1	2.128740365	RFX2	1.693490614
WNT7B	2.411615655	RGS22	1.741101141
XDH	1.866065983	RNF2	2.78948737
ZZEF1	1.647182035	RNF123	1.729074488

RPAP1	1.658639114
RPS6KA2	1.729074488
RSPO3	1.693490614
RUFY1	1.753211446
SCML2	2.027918933
SCN2A	1.958840634
SCN3B	1.647182067
SEMA3D	1.765405984
SERINC4	2.042024211
SETD1B	1.647182067
SHANK2	1.635804093
SLC43A2	1.658639114
SLIT1	1.67017585
SMAD1	1.635804093
SMG1	1.613283516
SNED1	1.67017585
SPATA13	1.635804093
ST6GALNAC5	1.658639114
STAB2	1.790050109
SUPT3H	1.67017585
SYNE1	1.635804093
TBC1D9	2.114036144
TCF12	1.647182067
TCP11L2	1.658639114
TEF	1.802500955
TGFBR3	1.67017585
THADA	1.635804093
TIAM1	1.647182067
TIAM2	1.741101141
TLL1	1.658639114
TLL2	1.753211446
TNRC6C	1.602139763
TNRC18	1.647182067
TP53I11	1.827662906
TRPV1	1.67017585
TTC21B	1.693490614
ULK4	1.729074488

UNC5C	1.717130839
UNC80	1.681792831
USH1C	1.790050109
USH2A	1.693490614
USP34	1.905276006
UTRN	1.635804093
VAC14	1.67017585
VPS13C	1.693490614
VSP13D	1.624504779
WASL	1.635804093
WDR19	1.777685342
WDR52	1.815038329
WDR69	1.741101141
WIF1	1.717130839
WIPF3	1.777685342
XRN1	1.67017585
YTHDC1	1.918528235
ZC3H7B	1.681792831
ZFC3H1	1.790050109
ZFP704	1.658639114
ZFYVE26	1.624504779
ZMIZ1	1.681792831
ZMYND8	1.658639114
ZNF236	1.635804093
ZNF384	1.602139763

B

Fold change	Up-regulated	Down-regulated
> 3	3	0
2.5 - 3.0	7	2
2.0 - 2.5	37	7
1.8 - 2.0	61	33
1.6 - 1.8	111	239
