# Supplementary material for: Regulatory mechanisms of testosterone-stimulated song in the sensorimotor nucleus HVC of female songbirds
Source: BMC Neurosci. 2014 Dec 2;15:128. doi: 10.1186/s12868-014-0128-0 (PMC4261767; doi:10.1186/s12868-014-0128-0)
Supplement: Additional file 4: Table S2. — Genes that showed strong expression changes (≥2-fold) after testosterone treatment in the HVC of female European robins. Genes were assigned to functional categories according to information obtained from PubMed (http://www.ncbi.nlm.nih.gov/pubmed) and GenCards (http://www.genecards.org/). [file 12868_2014_128_MOESM4_ESM.pdf]

Functional category	Zebra finch transcripts			Human orthologous genes	
	Gene	Accession number	Fold change	Symbol	Examples of cellular function/role in brain tissue
Signal transduction	Membrane protein, palmitoylated 1, 55kDa	XM_002189094	+3.1	MPP1	cGMP signaling
Signal transduction	similar to Phosphatidylinositol 4-kinase, catalytic, alpha	XM_002195771	+2.9	PI4KA	PtdIns(4)P/PtdIns(4,5)P2 synthesis: membrane trafficking, PKC activation, calcium signaling
Signal transduction	similar to Phosphoinositide-3-kinase, class 2, alpha polypeptide	XM_002197749	+2.6	PIK3C2A	PtdIns(4)P/PtdIns(4,5)P2 synthesis: membrane trafficking, PKC activation, calcium signaling
Signal transduction	similar to Dual specificity phosphatase 5	XM_002197720	+2.3	DUSP5	MAPK pathway
Signal transduction	Sperm associated antigen 9	XM_002198144	+2.3	SPAG9	MAPK pathway
Signal transduction	similar to GDNF family receptor alpha 1	XR_053899	+2.2	GFRA1	GDNF-receptor: neuron differentiation, synaptogenesis
Signal transduction	similar to Alpha keratin 1	XM_002194173	+2.2	KRT1	ECM-signaling: cell migration
Signal transduction	Mitogen-activated protein kinase 6	XM_002198097	+2.2	MAPK6	MAPK pathway
Signal transduction	similar to Mitogen-activated protein kinase 13	XM_002198912	+2.2	MAPK13	MAPK pathway
Signal transduction	similar to ATPase, Ca++ transporting, ubiquitous	XR_054375	+2.1	ATP2A3	Calcium sequestration into ER
Signal transduction	Ectodysplasin A2 receptor	XM_002189351	+2.1	EDA2R	EDA-receptor: pro-apoptotic
Signal transduction	Kinase insert domain receptor (a type III receptor tyrosine kinase)	XM_002195714	+2.1	KDR	VEGF receptor: angiogenesis
Signal transduction	similar to Docking protein 7	XM_002195142	+2.0	DOK7	Tyrosine kinase signaling
Signal transduction	similar to leucine-rich repeat kinase 1	XM_002197914	+2.0	LRRK1	MAPK pathway
Signal transduction	similar to Phosphodiesterase 4D, cAMP-specific	XM_002187817	+2.0	PDE4D	cAMP signaling
Signal transduction	similar to Phosphodiesterase 5A, cGMP-specific	XM_002198904	+2.0	PDE5A	cGMP signaling
Signal transduction	similar to thyrotropin-releasing hormone receptor 3	XM_002190995	+2.0	TRHR	TRH-receptor: neuromodulation
Signal transduction	similar to Serine incorporator 4	XM_002187749	-2.0	SERINC4	Lipogenesis: phosphatidylserine (PKC activation), sphingolipids (metabolite signaling)
Signal transduction	Leucine rich repeat containing 7	ENSTGUT00000010780	-2.6	LRRC7	Activity-dependent calcium signaling
Cellular homeostasis	LMBR1 domain containing 1	XM_002191722	+3.1	LMBRD1	Cobalamin coenzyme synthesis: cytokine homeostasis
Cellular homeostasis	Corticotropin releasing hormone binding protein	XM_002190644	+2.9	CRHBP	Energy homeostasis
Cellular homeostasis	similar to Hect domain and RLD 2	XM_001109970	+2.7	HERC2	Regulator of ubiquitylation signaling: e.g. DNA damage repair
Cellular homeostasis	ATPase, H+ transporting, lysosomal 42kDa, V1 subunit C1	XM_002198994	+2.3	ATP6V1C1	Regulation of cytosolic pH
Cellular homeostasis	similar to DnaJ (Hsp40) homolog, subfamily B, member 5	XM_002189924	+2.3	DNAJB5	Molecular chaperone
Cellular homeostasis	Deiodinase, iodothyronine, type II	ENSTGUT00000012872	+2.3	DIO2	Local thyroid hormone homeostasis
Cellular homeostasis	Urotensin 2 domain containing	XM_002190533	+2.2	UTS2D	Hypotension
Cellular homeostasis	Bombesin-like receptor 3	XM_002189155	+2.1	BRS3	Energy homeostasis: increased metabolic rate
Cellular homeostasis	similar to Fanconi anemia, complementation group A	XM_002195957	+2.1	FANCA	Inhibition of SPTAN1-protein cleavage: DNA damage repair
Cellular homeostasis	Coagulation factor XIII, A1 polypeptide	XM_002199749	+2.0	F13A1	Transglutaminase: crosslinking to fibrin in the ECM
Cellular homeostasis	Neuropeptide Y	XM_002193012	+2.0	NPY	Energy homeostasis; Modulation of neurotransmitter release and sensitivity; Angiogenesis; Neurogenesis;
Cellular homeostasis	similar to solute carrier family 6, member 17	ENSTGUG00000000875	+2.0	SLC6A17	Glutamate and GABA re-uptake
Cellular homeostasis	Solute carrier family 40 (iron-regulated transporter), member 1	XM_002191905	+2.0	SLC40A1	Iron export
Cellular homeostasis	Melanocortin 4 receptor	XM_002199220	-2.0	MC4R	Leptin-dependent energy homeostasis: increased metabolic rate
Cell-Cell/ECM interaction	Collagen, type VI, alpha 1	XM_002190829	+2.6	COL6A1	ECM: neuron survival
Cell-Cell/ECM interaction	odz, odd Oz/ten-m homolog 1(Drosophila)	XM_002197079	+2.5	ODZ1	Formation of neuronal connections

	similar to WAP four-disulfide core domain 1	XM_002194482	+2.1	WFDC1	ECM-modulation: pro-angiogenic
	Collagen, type IV, alpha 1	XM_002190412	+2.0	COL4A1	Basement membrane: brain development
	similar to Dermatopontin	XM_002193636		DPT	ECM: collagen fibrillogenesis, cell anchoring
	Endomucin	ENSTGUT00000003661		EMCN	Endothelial cells: anti-adhesive
	Multimerin-2 Precursor	ENSTGUT00000005954		MMRN2	Vascular endothelial cell surface
	similar to Otogelin-like protein C12orf64	XM_002191580	-2.0	OTOGL	ECM
	FAT tumor suppressor homolog 3	XM_002200159	-2.2	FAT3	Neurite interaction
Cellular metabolism	Fatty acid desaturase 2	XM_002194908	+3.1	FADS2	Long-chain polyunsaturated membrane phospholipid synthesis
	Kinectin 1	XM_002200392	+2.5	KTN1	Endoplasmic reticulum mobility; Membrane protein synthesis
	Aldehyde dehydrogenase 1 family, member A2	NM_001076685	+2.2	ALDH1A2	Retinoic acid synthesis: bird song maturation
	Acyl-CoA synthetase bubblegum family member 2	ENSTGUT00000000379	+2.0	ACSBG2	Fatty acid metabolism
	similar to GalNAc-4-sulfotransferase 2	XM_002200243		CHST9	Sulfation of glycoproteins
	TBC1 domain family, member 9 (with GRAM domain)	XM_002192083	-2.1	TBC1D9	Rab-GAB: inactivation of a specific membrane trafficking event
	Ecto-NOX disulfide-thiol exchanger 1	XM_002196081	-2.2	ENOX1	Mitosis: cell enlargement
Growth factors	Tumor necrosis factor ligand superfamily member 13B	ENSTGUT00000011241	+2.9	TNFSF13B	Development of humoral immune response; Inhibition of neurite outgrowth
	similar to wingless-type MMTV integration site family, member 7B	XM_002187951	+2.4	WNT7B	Angiogenesis; Dendritic arborization
	Brain-derived neurotrophic factor	NM_001048255	+2.2	BDNF	Bird song maturation (see text)
	similar to Placental growth factor	XM_002199929		PGF	Angiogenesis; Neuron survival
	Calcium-dependent secretion activator 2	ENSTGUT00000004647	+2.0	CADPS2	BDNF release
	Insulin-like growth factor 2	XM_002186502		IGF2	Bird song nuclei development
	Met proto-oncogene	XM_002193399		MET	HGF-receptor: pro-angiogenic
Ion channels and neurotransmitter release	similar to Synaptic vesicle glycoprotein 2b	XM_002197697	+2.3	SV2B	Neurotransmitter release
	Ovoinhibitor	XM_002194425	+2.1	SPINK5	Inhibition of BK channels: feedback on VGCC opening
	similar to Ras like G protein Rad	ENSTGUT00000010101	+2.0	RRAD	Regulator of VGCC activity: Neurotransmitter release
	similar to Dmx-like 2	XM_002196449	-2.0	DMXL2	Rab3 GDP/GTP exchange: regulation of neurotransmittter release
	sodium channel, voltage-gated, type II, alpha subunit	ENSTGUT00000007427		SCN2A	Excitability
	Peroxisomal biogenesis factor 5-like	XM_002193042	-2.2	PEX5L	Regulation of surface expression and inhibition of HCN1 channels: excitability, rhythmic activity, dendritic integration
Cytoskeleton	similar to Actinin, alpha 2	XM_002195531	+2.4	ACTN2	Membrane protein localization
	similar to Myosin X	XM_002193143		MYO10	Cytoskeletal dynamics
	Spectrin, alpha, non-erythrocytic 1 (alpha-fodrin)	XM_002197783	+2.2	SPTAN1	Cytoskeleton organization; Mitosis and DNA repair
	Transmembrane protein 201	XM_002187261		TMEM201	Mitosis
	Neurofilament medium polypeptide	XM_002197502	+2.1	NEFM	Neuronal development
	Microtubule-actin crosslinking factor 1	ENSTGUT00000001370	+2.0	MACF1	Cytoskeletal dynamics: cell migration
Transcription regulation	Single stranded DNA binding protein 3	XR_054511	+2.4	SSBP3	Neuron development
	Androgen receptor	NM_001076688	+2.0	AR	Bird song maturation
	similar to Snail homolog 2 (Drosophila)	XM_002197191		SNAI2	Gene repression: pro cell migration
	similar to Mesoderm induction early response 1, family member 2	XM_002193165	-2.0	MIER2	Gene repression (ESR1-corepressor)
	Sex comb on midleg-like 2 (Drosophila)	XM_002196976		SCML2	PcG1 subunits: neural stem cell proliferation, repression of neurogenic genes and neurogenic to astrogenic fate switch
	similar to Ring finger protein 2	XM_002190830	-2.8	RNF2	
