# Supplementary material for: Regulatory mechanisms of testosterone-stimulated song in the sensorimotor nucleus HVC of female songbirds
Source: BMC Neurosci. 2014 Dec 2;15:128. doi: 10.1186/s12868-014-0128-0 (PMC4261767; doi:10.1186/s12868-014-0128-0)
Supplement: Additional file 6: Figure S4. — Genes that showed expression changes after testosterone treatment and are expected to play a role for the blood vessel development in the HVC of female European robins. (A) The largest network of up-regulated genes that we discovered by GePS (Genomatix) analysis included two angiogenic ligands (VEGFA, PGF). Further, (B) an association with angiogenic processes has been reported for most of the other genes of this network. [file 12868_2014_128_MOESM6_ESM.pdf]

A

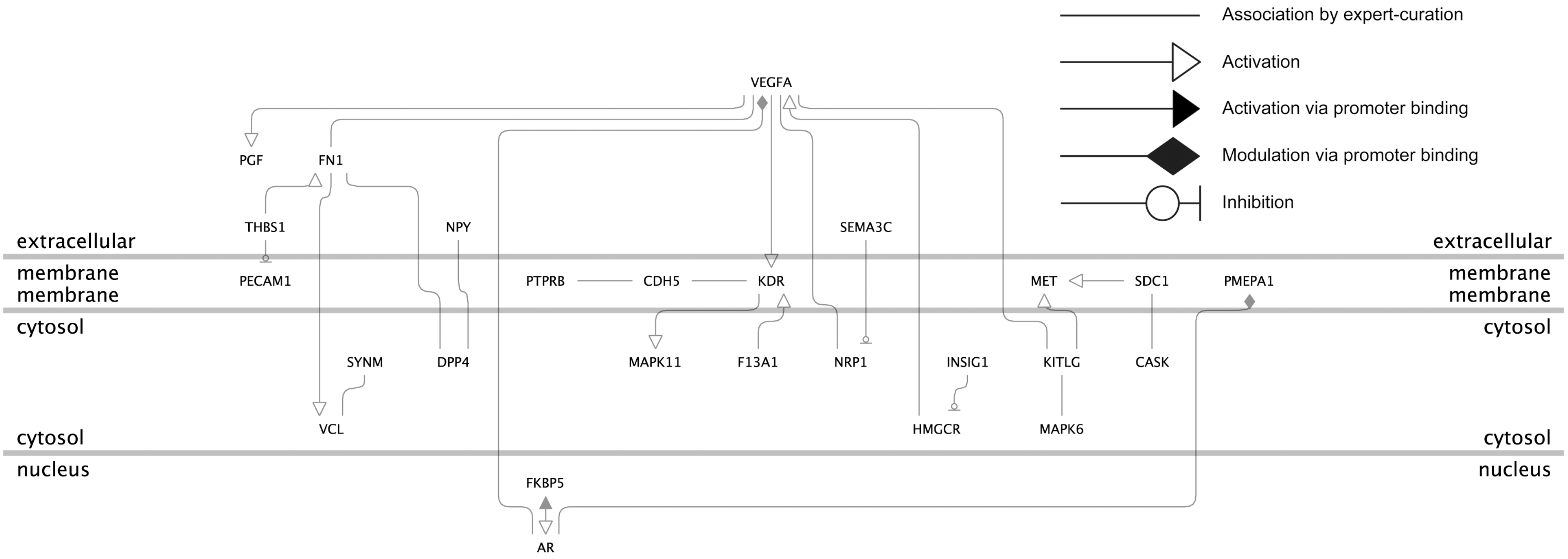

B

Symbol	Gene name	Reference
AR	androgen receptor	
CASK	calcium/calmodulin-dependent serine protein kinase	
CDH5	cadherin 5, type 2	2
DPP4	dipeptidyl-peptidase 4	5
F13A1	coagulation factor XIII, A1 polypeptide	
FKBP5	FK506 binding protein 5	
FN1	fibronectin 1	12
HMGCR	3-hydroxy-3-methylglutaryl-CoA reductase	3
INSIG1	insulin induced gene 1	
KDR	kinase insert domain receptor	10
KITLG	KIT ligand	11
MAPK6	mitogen-activated protein kinase 6	
MAPK11	mitogen-activated protein kinase 11	
MET	met proto-oncogene	1
NPY	neuropeptide Y	4
NRP1	neuropilin 1	10
PECAM1	platelet/endothelial cell adhesion molecule 1	9
PGF	placental growth factor	10
PMEPA1	prostate transmembrane protein, androgen induced 1	
PTPRB	protein tyrosine phosphatase, receptor type, B	6
SDC1	syndecan 1	7
SEMA3C	sema domain, immunoglobulin domain (Ig), short basic domain, secreted, (semaphorin) 3C	8
SYNM	synemin, intermediate filament protein	
THBS1	thrombospondin 1	9
VEGFA	vascular endothelial growth factor A	10
VCL	vinculin	

References:

[1]Abounader R, Laterra J: **Scatter factor/hepatocyte growth factor in brain tumor growth and angiogenesis.** *Neuro Oncol* 2005, 7:436-451.

[2]Carmeliet P, Lampugnani MG, Moons L, Breviario F, Compernolle V, Bono F, Balconi G, Spagnuolo R, Oosthuyse B, Dewerchin M, Zanetti A, Angellilo A, Mattot V, Nuyens D, Lutgens E, Clotman F, de Ruiter MC, Gittenberger-de Groot A, Poelmann R, Lupu F, Herbert JM, Collen D, Dejana E: **Targeted deficiency or cytosolic truncation of the VE-cadherin gene in mice impairs VEGF-mediated endothelial survival and angiogenesis.** *Cell* 1999, 98:147-157.

[3]Eisa-Beygi S, Hatch G, Noble S, Ekker M, Moon TW: **The 3-hydroxy-3-methylglutaryl-CoA reductase (HMGCR) pathway regulates developmental cerebral-vascular stability via prenylation-dependent signalling pathway.** *Dev Biol* 2013, 373:258-266.

[4]Ekstrand AJ, Cao R, Bjorndahl M, Nystrom S, Jonsson-Rylander AC, Hassani H, Hallberg B, Nordlander M, Cao Y: **Deletion of neuropeptide Y (NPY) 2 receptor in mice results in blockage of NPY-induced angiogenesis and delayed wound healing.** *Proc Natl Acad Sci U S A* 2003, 100:6033-6038.

[5]Kitlinska J, Lee EW, Li L, Pons J, Estes L, Zukowska Z: **Dual role of dipeptidyl peptidase IV (DPP IV) in angiogenesis and vascular remodeling.** *Adv Exp Med Biol* 2003, 524:215-222.

[6]Mori M, Murata Y, Kotani T, Kusakari S, Ohnishi H, Saito Y, Okazawa H, Ishizuka T, Mori M, Matozaki T: **Promotion of cell spreading and migration by vascular endothelial-protein tyrosine phosphatase (VE-PTP) in cooperation with integrins.** *J Cell Physiol* 2010, 224:195-204.

[7]Rapraeger AC, Ell BJ, Roy M, Li X, Morrison OR, Thomas GM, Beauvais DM: **Vascular endothelial-cadherin stimulates syndecan-1-coupled insulin-like growth factor-1 receptor and cross-talk between αVβ3 integrin and vascular endothelial growth factor receptor 2 at the onset of endothelial cell dissemination during angiogenesis.** *FEBS J* 2013, 280:2194-2206.

[8]Salikhova A, Wang L, Lanahan AA, Liu M, Simons M, Leenders WP, Mukhopadhyay D, Horowitz A: **Vascular endothelial growth factor and semaphorin induce neuropilin-1 endocytosis via separate pathways.** *Circ Res* 2008, 103:e71-79.

[9]Sheibani N, Frazier WA: **Thrombospondin-1, PECAM-1, and regulation of angiogenesis.** *Histol Histopathol* 1999, 14:285-294.

[10]Shibuya M: **Vascular endothelial growth factor and its receptor system: physiological functions in angiogenesis and pathological roles in various diseases.** *J Biochem* 2013, 153:13-19.

[11]Sun L, Hui AM, Su Q, Vortmeyer A, Kotliarov Y, Pastorino S, Passaniti A, Menon J, Walling J, Bailey R, Rosenblum M, Mikkelsen T, Fine HA: **Neuronal and glioma-derived stem cell factor induces angiogenesis within the brain.** *Cancer Cell* 2006, 9:287-300.

[12]Wang J, Milner R: **Fibronectin promotes brain capillary endothelial cell survival and proliferation through alpha5beta1 and alphavbeta3 integrins via MAP kinase signalling.** *J Neurochem* 2006, 96:148-159.
