# Supplementary material for: Regulatory mechanisms of testosterone-stimulated song in the sensorimotor nucleus HVC of female songbirds
Source: BMC Neurosci. 2014 Dec 2;15:128. doi: 10.1186/s12868-014-0128-0 (PMC4261767; doi:10.1186/s12868-014-0128-0)
Supplement: Additional file 7: Table S3. — Enrichment of biological processes in genes that showed expression changes after testosterone treatment in the HVC of female European robins and canaries. Probabilities of biological processes enriched in (A, B) up- and (C, D) down-regulated genes expressed in the HVC of robins (A, C) and canaries (B, D), respectively, were determined by GeneRanker (Genomatix). For (E) explicitly neuronal GO-terms were selected from the list of genes that were down-regulated in the HVC of female robins genes and grouped as shown for up-regulated genes in Figure 4 of the main text. For comparison probabilities of the same processes are also shown for female canaries. Enrichment of neuron projection development, neuron development/differentiation and neurogenesis in both up- and down-regulated genes suggested that these processes were dynamically affected following the testosterone treatment. [file 12868_2014_128_MOESM7_ESM.pdf]

A

Accession	GO Term Biological Process enriched in up regulated genes	Robin
GO:0001568	blood vessel development	8.58E-10
GO:0001944	vasculature development	2.41E-09
GO:0040011	locomotion	4.53E-08
GO:0048856	anatomical structure development	2.08E-07
GO:0072358	cardiovascular system development	3.26E-07
GO:0072359	circulatory system development	3.26E-07
GO:0009605	response to external stimulus	9.15E-07
GO:0050896	response to stimulus	9.18E-07
GO:0042221	response to chemical stimulus	1.21E-06
GO:0006695	cholesterol biosynthetic process	1.28E-06
GO:0006935	chemotaxis	1.47E-06
GO:0042330	taxis	1.47E-06
GO:0032502	developmental process	2.00E-06
GO:0048646	anatomical structure formation involved in morphogenesis	2.07E-06
GO:0007411	axon guidance	2.74E-06
GO:0007275	multicellular organismal development	3.09E-06
GO:0016126	sterol biosynthetic process	3.19E-06
GO:0048731	system development	3.22E-06
GO:0032501	multicellular organismal process	3.41E-06
GO:0008299	isoprenoid biosynthetic process	8.02E-06
GO:0007155	cell adhesion	8.44E-06
GO:0022610	biological adhesion	8.44E-06
GO:0045636	positive regulation of melanocyte differentiation	8.72E-06
GO:0050942	positive regulation of pigment cell differentiation	8.72E-06
GO:0072089	stem cell proliferation	8.74E-06
GO:0048583	regulation of response to stimulus	9.92E-06
GO:0009653	anatomical structure morphogenesis	1.04E-05
GO:0002576	platelet degranulation	1.16E-05
GO:0006694	steroid biosynthetic process	1.36E-05
GO:0030154	cell differentiation	1.42E-05
GO:0048514	blood vessel morphogenesis	1.70E-05

GO:0032101	regulation of response to external stimulus	1.93E-05
GO:0048087	positive regulation of developmental pigmentation	2.17E-05
GO:0007409	axonogenesis	2.67E-05
GO:0001525	angiogenesis	2.98E-05
GO:0000904	cell morphogenesis involved in differentiation	3.24E-05
GO:0006720	isoprenoid metabolic process	3.53E-05
GO:0023052	signaling	3.59E-05
GO:0048869	cellular developmental process	3.87E-05
GO:0045634	regulation of melanocyte differentiation	4.29E-05
GO:0050932	regulation of pigment cell differentiation	4.29E-05
GO:0043405	regulation of MAP kinase activity	4.31E-05
GO:0035295	tube development	4.76E-05
GO:0060429	epithelium development	4.83E-05
GO:0009888	tissue development	5.51E-05
GO:0048468	cell development	6.71E-05
GO:0008283	cell proliferation	7.14E-05
GO:0048667	cell morphogenesis involved in neuron differentiation	8.01E-05
GO:0008610	lipid biosynthetic process	8.78E-05
GO:0048812	neuron projection morphogenesis	9.05E-05
GO:0007154	cell communication	9.07E-05
GO:0031175	neuron projection development	9.17E-05
GO:0030324	lung development	9.38E-05
GO:0016477	cell migration	9.61E-05
GO:0045137	development of primary sexual characteristics	9.76E-05
GO:0023051	regulation of signaling	1.08E-04
GO:0030323	respiratory tube development	1.10E-04
GO:0051592	response to calcium ion	1.14E-04
GO:0009790	embryo development	1.28E-04
GO:0042127	regulation of cell proliferation	1.29E-04
GO:0006887	exocytosis	1.47E-04
GO:0030168	platelet activation	1.48E-04
GO:0060449	bud elongation involved in lung branching	1.70E-04
GO:0008406	gonad development	1.94E-04
GO:0048666	neuron development	2.09E-04

GO:0031589	cell-substrate adhesion	2.11E-04
GO:0032990	cell part morphogenesis	2.29E-04
GO:0051716	cellular response to stimulus	2.31E-04
GO:0003158	endothelium development	2.34E-04
GO:0030855	epithelial cell differentiation	2.57E-04
GO:0000165	MAPK cascade	2.61E-04
GO:0007165	signal transduction	2.64E-04
GO:0032989	cellular component morphogenesis	2.67E-04
GO:0009611	response to wounding	2.69E-04
GO:0010038	response to metal ion	2.72E-04
GO:0032103	positive regulation of response to external stimulus	2.79E-04
GO:0048870	cell motility	2.86E-04
GO:0051674	localization of cell	2.86E-04
GO:0007548	sex differentiation	2.93E-04
GO:0060541	respiratory system development	2.96E-04
GO:0007243	intracellular protein kinase cascade	2.99E-04
GO:0001936	regulation of endothelial cell proliferation	3.04E-04
GO:0000902	cell morphogenesis	3.05E-04
GO:0048608	reproductive structure development	3.14E-04
GO:0030155	regulation of cell adhesion	3.26E-04
GO:0030198	extracellular matrix organization	3.27E-04
GO:0043062	extracellular structure organization	3.27E-04
GO:0050793	regulation of developmental process	3.31E-04
GO:0007160	cell-matrix adhesion	4.21E-04
GO:0006928	cellular component movement	4.25E-04
GO:0030334	regulation of cell migration	4.37E-04
GO:0043408	regulation of MAPK cascade	4.45E-04
GO:0048070	regulation of developmental pigmentation	4.45E-04
GO:0071900	regulation of protein serine-threonine kinase activity	4.63E-04
GO:0010627	regulation of intracellular protein kinase cascade	4.70E-04
GO:0045765	regulation of angiogenesis	4.87E-04
GO:0048858	cell projection morphogenesis	5.19E-04
GO:0009966	regulation of signal transduction	5.32E-04
GO:0008203	cholesterol metabolic process	5.40E-04

GO:0050673	epithelial cell proliferation	5.42E-04
GO:0071363	cellular response to growth factor stimulus	5.43E-04
GO:0042981	regulation of apoptotic process	5.55E-04
GO:0035556	intracellular signal transduction	5.62E-04
GO:0030030	cell projection organization	5.69E-04
GO:0046546	development of primary male sexual characteristics	5.71E-04
GO:0014032	neural crest cell development	5.76E-04
GO:0034330	cell junction organization	6.11E-04
GO:0043067	regulation of programmed cell death	6.28E-04
GO:0003006	developmental process involved in reproduction	6.29E-04
GO:0030182	neuron differentiation	6.41E-04
GO:0010035	response to inorganic substance	6.44E-04
GO:0048754	branching morphogenesis of a tube	6.44E-04
GO:0001763	morphogenesis of a branching structure	6.61E-04
GO:0050678	regulation of epithelial cell proliferation	6.88E-04
GO:0001935	endothelial cell proliferation	7.14E-04
GO:2000145	regulation of cell motility	7.17E-04
GO:0051234	establishment of localization	7.63E-04
GO:0016125	sterol metabolic process	7.86E-04
GO:0009719	response to endogenous stimulus	8.26E-04
GO:0046661	male sex differentiation	8.28E-04
GO:0006810	transport	8.52E-04
GO:0022008	neurogenesis	8.78E-04
GO:0042692	muscle cell differentiation	8.81E-04
GO:0010941	regulation of cell death	9.38E-04
GO:0048519	negative regulation of biological process	9.41E-04
GO:0007169	transmembrane receptor protein tyrosine kinase signaling pathway	9.50E-04
GO:0010810	regulation of cell-substrate adhesion	9.76E-04
GO:0070848	response to growth factor stimulus	9.82E-04
GO:0060572	morphogenesis of an epithelial bud	1.09E-03
GO:0048584	positive regulation of response to stimulus	1.10E-03
GO:0014033	neural crest cell differentiation	1.11E-03
GO:0050795	regulation of behavior	1.11E-03
GO:0065007	biological regulation	1.16E-03

GO:0022603	regulation of anatomical structure morphogenesis	1.18E-03
GO:0051179	localization	1.21E-03
GO:0050920	regulation of chemotaxis	1.23E-03
GO:0051270	regulation of cellular component movement	1.28E-03
GO:0065008	regulation of biological quality	1.28E-03
GO:0035924	cellular response to vascular endothelial growth factor stimulus	1.31E-03
GO:0034329	cell junction assembly	1.32E-03
GO:0071902	positive regulation of protein serine-threonine kinase activity	1.33E-03
GO:0048041	focal adhesion assembly	1.34E-03
GO:0043066	negative regulation of apoptotic process	1.38E-03
GO:0043406	positive regulation of MAP kinase activity	1.38E-03
GO:0043542	endothelial cell migration	1.46E-03
GO:0040012	regulation of locomotion	1.48E-03
GO:0045446	endothelial cell differentiation	1.48E-03
GO:0043069	negative regulation of programmed cell death	1.51E-03
GO:0008584	male gonad development	1.54E-03
GO:0001885	endothelial cell development	1.56E-03
GO:0034220	ion transmembrane transport	1.56E-03
GO:0010595	positive regulation of endothelial cell migration	1.64E-03
GO:0032940	secretion by cell	1.64E-03
GO:0010839	negative regulation of keratinocyte proliferation	1.66E-03
GO:0048012	hepatocyte growth factor receptor signaling pathway	1.66E-03
GO:0060666	dichotomous subdivision of terminal units involved in salivary gland branching	1.66E-03
GO:0006629	lipid metabolic process	1.76E-03
GO:0007267	cell-cell signaling	1.81E-03
GO:0048699	generation of neurons	1.90E-03
GO:0051094	positive regulation of developmental process	1.91E-03
GO:0010033	response to organic substance	2.02E-03
GO:0035162	embryonic hemopoiesis	2.14E-03
GO:0040036	regulation of fibroblast growth factor receptor signaling pathway	2.14E-03
GO:0051893	regulation of focal adhesion assembly	2.14E-03
GO:0060445	branching involved in salivary gland morphogenesis	2.14E-03
GO:0090109	regulation of cell-substrate junction assembly	2.14E-03
GO:0044255	cellular lipid metabolic process	2.22E-03

GO:0060548	negative regulation of cell death	2.24E-03
GO:0061061	muscle structure development	2.27E-03
GO:0048010	vascular endothelial growth factor receptor signaling pathway	2.36E-03
GO:0048513	organ development	2.41E-03
GO:0010463	mesenchymal cell proliferation	2.57E-03
GO:0007399	nervous system development	2.70E-03
GO:0046903	secretion	2.76E-03
GO:0060485	mesenchyme development	2.76E-03
GO:0010811	positive regulation of cell-substrate adhesion	2.79E-03
GO:0008202	steroid metabolic process	2.83E-03
GO:0045766	positive regulation of angiogenesis	2.83E-03
GO:0010646	regulation of cell communication	2.86E-03
GO:0042060	wound healing	2.99E-03
GO:0016310	phosphorylation	3.01E-03
GO:0010453	regulation of cell fate commitment	3.22E-03
GO:0021846	cell proliferation in forebrain	3.22E-03
GO:0050789	regulation of biological process	3.22E-03
GO:0060571	morphogenesis of an epithelial fold	3.22E-03
GO:0007215	glutamate receptor signaling pathway	3.27E-03
GO:0048523	negative regulation of cellular process	3.28E-03
GO:0006950	response to stress	3.38E-03
GO:0038084	vascular endothelial growth factor signaling pathway	3.43E-03
GO:0060592	mammary gland formation	3.43E-03
GO:0072091	regulation of stem cell proliferation	3.43E-03
GO:2000310	regulation of N-methyl-D-aspartate selective glutamate receptor activity	3.43E-03
GO:0000188	inactivation of MAPK activity	3.65E-03
GO:0042445	hormone metabolic process	3.88E-03
GO:0042493	response to drug	3.91E-03
GO:0008285	negative regulation of cell proliferation	3.95E-03
GO:0071495	cellular response to endogenous stimulus	4.06E-03
GO:0006468	protein phosphorylation	4.09E-03
GO:0050794	regulation of cellular process	4.10E-03
GO:0061138	morphogenesis of a branching epithelium	4.16E-03
GO:0009791	post-embryonic development	4.18E-03

GO:0035107	appendage morphogenesis	4.20E-03
GO:0035108	limb morphogenesis	4.20E-03
GO:0051403	stress-activated MAPK cascade	4.31E-03
GO:0008343	adult feeding behavior	4.53E-03
GO:0035767	endothelial cell chemotaxis	4.53E-03
GO:0060484	lung-associated mesenchyme development	4.53E-03
GO:0061028	establishment of endothelial barrier	4.53E-03
GO:0050878	regulation of body fluid levels	4.59E-03
GO:0001952	regulation of cell-matrix adhesion	4.72E-03
GO:0031098	stress-activated protein kinase signaling cascade	4.72E-03
GO:0003007	heart morphogenesis	4.79E-03
GO:0001101	response to acid	4.89E-03
GO:0043410	positive regulation of MAPK cascade	4.96E-03
GO:0007044	cell-substrate junction assembly	5.05E-03
GO:0010740	positive regulation of intracellular protein kinase cascade	5.11E-03
GO:0018108	peptidyl-tyrosine phosphorylation	5.13E-03
GO:0030879	mammary gland development	5.37E-03
GO:0002688	regulation of leukocyte chemotaxis	5.40E-03
GO:0070887	cellular response to chemical stimulus	5.42E-03
GO:0018212	peptidyl-tyrosine modification	5.48E-03
GO:0048762	mesenchymal cell differentiation	5.58E-03
GO:0006200	ATP catabolic process	5.67E-03
GO:0018149	peptide cross-linking	5.68E-03
GO:0035115	embryonic forelimb morphogenesis	5.68E-03
GO:0043009	chordate embryonic development	5.72E-03
GO:0042574	retinal metabolic process	5.78E-03
GO:0043288	apocarotenoid metabolic process	5.78E-03
GO:0060536	cartilage morphogenesis	5.78E-03
GO:0060841	venous blood vessel development	5.78E-03
GO:0071392	cellular response to estradiol stimulus	5.78E-03
GO:0007596	blood coagulation	6.16E-03
GO:0000910	cytokinesis	6.24E-03
GO:0007435	salivary gland morphogenesis	6.28E-03
GO:0048008	platelet-derived growth factor receptor signaling pathway	6.28E-03

GO:0060428	lung epithelium development	6.28E-03
GO:0048729	tissue morphogenesis	6.36E-03
GO:0009792	embryo development ending in birth or egg hatching	6.49E-03
GO:0045859	regulation of protein kinase activity	6.58E-03
GO:0007599	hemostasis	6.59E-03
GO:0050817	coagulation	6.59E-03
GO:0048732	gland development	6.65E-03
GO:0007610	behavior	6.73E-03
GO:0048736	appendage development	6.75E-03
GO:0060173	limb development	6.75E-03
GO:0002009	morphogenesis of an epithelium	6.81E-03
GO:0060711	labyrinthine layer development	6.91E-03
GO:0060326	cell chemotaxis	7.01E-03
GO:0015936	coenzyme A metabolic process	7.16E-03
GO:0060174	limb bud formation	7.16E-03
GO:0046034	ATP metabolic process	7.27E-03
GO:2000026	regulation of multicellular organismal development	7.29E-03
GO:0051781	positive regulation of cell division	7.39E-03
GO:0001657	ureteric bud development	7.49E-03
GO:0007163	establishment or maintenance of cell polarity	7.49E-03
GO:0006915	apoptotic process	7.53E-03
GO:0002053	positive regulation of mesenchymal cell proliferation	7.58E-03
GO:0031399	regulation of protein modification process	7.78E-03
GO:0008360	regulation of cell shape	7.83E-03
GO:0001570	vasculogenesis	7.84E-03
GO:0043407	negative regulation of MAP kinase activity	7.84E-03
GO:0001701	in utero embryonic development	8.06E-03
GO:0050731	positive regulation of peptidyl-tyrosine phosphorylation	8.18E-03
GO:0006916	anti-apoptosis	8.25E-03
GO:0030318	melanocyte differentiation	8.28E-03
GO:0010594	regulation of endothelial cell migration	8.31E-03
GO:0012501	programmed cell death	8.40E-03
GO:0008354	germ cell migration	8.67E-03
GO:0009214	cyclic nucleotide catabolic process	8.67E-03

GO:0010837	regulation of keratinocyte proliferation	8.67E-03
GO:0032793	positive regulation of CREB transcription factor activity	8.67E-03
GO:0060736	prostate gland growth	8.67E-03
GO:0071622	regulation of granulocyte chemotaxis	8.67E-03
GO:0080134	regulation of response to stress	8.70E-03
GO:0003281	ventricular septum development	9.03E-03
GO:0007431	salivary gland development	9.03E-03
GO:0050931	pigment cell differentiation	9.03E-03
GO:0060603	mammary gland duct morphogenesis	9.03E-03
GO:0061180	mammary gland epithelium development	9.29E-03
GO:0022612	gland morphogenesis	9.68E-03
GO:0046545	development of primary female sexual characteristics	9.68E-03
GO:0035136	forelimb morphogenesis	9.81E-03
GO:0043200	response to amino acid stimulus	9.81E-03
GO:0008219	cell death	9.96E-03
GO:0045860	positive regulation of protein kinase activity	9.96E-03

B

Accession	GO Term Biological Process enriched in up regulated genes	Canary
GO:0023052	signaling	2.63E-18
GO:0007154	cell communication	9.71E-18
GO:0032501	multicellular organismal process	3.57E-15
GO:0051179	localization	7.31E-14
GO:0007275	multicellular organismal development	7.14E-13
GO:0044281	small molecule metabolic process	2.44E-12
GO:0048731	system development	4.76E-12
GO:0040011	locomotion	1.09E-11

GO:0006928	cellular component movement	1.82E-11
GO:0007165	signal transduction	1.93E-11
GO:0032502	developmental process	1.84E-10
GO:0048856	anatomical structure development	2.02E-10
GO:0048666	neuron development	2.20E-10
GO:0050896	response to stimulus	3.36E-10
GO:0048667	cell morphogenesis involved in neuron differentiation	4.03E-10
GO:0016477	cell migration	4.49E-10
GO:0051716	cellular response to stimulus	5.24E-10
GO:0048812	neuron projection morphogenesis	5.90E-10
GO:0031175	neuron projection development	7.50E-10
GO:0023051	regulation of signaling	7.62E-10
GO:0042221	response to chemical stimulus	8.69E-10
GO:0048870	cell motility	1.18E-09
GO:0051674	localization of cell	1.18E-09
GO:0048468	cell development	1.69E-09
GO:0007399	nervous system development	3.31E-09
GO:0051234	establishment of localization	4.47E-09
GO:0007409	axonogenesis	5.66E-09
GO:0009987	cellular process	7.74E-09
GO:0007267	cell-cell signaling	1.08E-08
GO:0000904	cell morphogenesis involved in differentiation	1.20E-08
GO:0035556	intracellular signal transduction	2.03E-08
GO:0035637	multicellular organismal signaling	2.41E-08
GO:0022008	neurogenesis	2.65E-08
GO:0030182	neuron differentiation	2.88E-08
GO:0006810	transport	2.93E-08
GO:0048699	generation of neurons	2.96E-08
GO:0030154	cell differentiation	3.71E-08
GO:0040012	regulation of locomotion	3.84E-08
GO:0019226	transmission of nerve impulse	4.56E-08
GO:0003008	system process	5.69E-08
GO:0030030	cell projection organization	1.09E-07
GO:0007268	synaptic transmission	1.19E-07

GO:0048858	cell projection morphogenesis	1.20E-07
GO:0030334	regulation of cell migration	1.32E-07
GO:0032879	regulation of localization	1.36E-07
GO:0010035	response to inorganic substance	1.51E-07
GO:0051270	regulation of cellular component movement	1.74E-07
GO:0032990	cell part morphogenesis	2.01E-07
GO:2000145	regulation of cell motility	2.07E-07
GO:0048869	cellular developmental process	2.25E-07
GO:0055086	nucleobase-containing small molecule metabolic process	2.74E-07
GO:0046483	heterocycle metabolic process	3.38E-07
GO:0000902	cell morphogenesis	4.78E-07
GO:0032989	cellular component morphogenesis	4.85E-07
GO:0006753	nucleoside phosphate metabolic process	4.85E-07
GO:0009117	nucleotide metabolic process	4.85E-07
GO:0009605	response to external stimulus	7.48E-07
GO:0009966	regulation of signal transduction	8.01E-07
GO:0072521	purine-containing compound metabolic process	8.21E-07
GO:0007411	axon guidance	8.52E-07
GO:0009653	anatomical structure morphogenesis	1.00E-06
GO:0001666	response to hypoxia	1.81E-06
GO:0065007	biological regulation	1.85E-06
GO:0007169	transmembrane receptor protein tyrosine kinase signaling pathway	2.08E-06
GO:0006163	purine nucleotide metabolic process	2.23E-06
GO:0050877	neurological system process	3.99E-06
GO:0007166	cell surface receptor signaling pathway	4.43E-06
GO:0030155	regulation of cell adhesion	5.00E-06
GO:0007167	enzyme linked receptor protein signaling pathway	5.07E-06
GO:0008283	cell proliferation	5.54E-06
GO:0050793	regulation of developmental process	6.00E-06
GO:0050794	regulation of cellular process	6.82E-06
GO:0070482	response to oxygen levels	7.27E-06
GO:0000165	MAPK cascade	7.37E-06
GO:0006732	coenzyme metabolic process	7.94E-06
GO:0010038	response to metal ion	9.21E-06

GO:0006811	ion transport	1.16E-05
GO:0015980	energy derivation by oxidation of organic compounds	1.21E-05
GO:0006935	chemotaxis	1.37E-05
GO:0042330	taxis	1.37E-05
GO:0009060	aerobic respiration	1.42E-05
GO:0035338	long-chain fatty-acyl-CoA biosynthetic process	1.68E-05
GO:0042325	regulation of phosphorylation	1.99E-05
GO:0016043	cellular component organization	2.04E-05
GO:0071842	cellular component organization at cellular level	2.05E-05
GO:0010646	regulation of cell communication	2.14E-05
GO:0010033	response to organic substance	2.21E-05
GO:0006084	acetyl-CoA metabolic process	2.23E-05
GO:0034329	cell junction assembly	2.49E-05
GO:0050789	regulation of biological process	2.91E-05
GO:0030705	cytoskeleton-dependent intracellular transport	2.94E-05
GO:0007610	behavior	2.97E-05
GO:0007264	small GTPase mediated signal transduction	2.99E-05
GO:0006099	tricarboxylic acid cycle	3.00E-05
GO:0046356	acetyl-CoA catabolic process	3.00E-05
GO:0051239	regulation of multicellular organismal process	3.22E-05
GO:0046460	neutral lipid biosynthetic process	3.35E-05
GO:0046463	acylglycerol biosynthetic process	3.35E-05
GO:0048583	regulation of response to stimulus	3.41E-05
GO:0071841	cellular component organization or biogenesis at cellular level	3.65E-05
GO:0035336	long-chain fatty-acyl-CoA metabolic process	3.74E-05
GO:0046949	fatty-acyl-CoA biosynthetic process	3.74E-05
GO:0051338	regulation of transferase activity	3.75E-05
GO:0031589	cell-substrate adhesion	3.98E-05
GO:0009108	coenzyme biosynthetic process	4.26E-05
GO:0071840	cellular component organization or biogenesis	4.31E-05
GO:0034330	cell junction organization	4.39E-05
GO:0034220	ion transmembrane transport	4.44E-05
GO:0035384	thioester biosynthetic process	5.36E-05
GO:0071616	acyl-CoA biosynthetic process	5.36E-05

GO:0009259	ribonucleotide metabolic process	5.63E-05
GO:0065008	regulation of biological quality	5.88E-05
GO:0010970	microtubule-based transport	5.97E-05
GO:0001935	endothelial cell proliferation	6.12E-05
GO:0043549	regulation of kinase activity	6.62E-05
GO:0040013	negative regulation of locomotion	6.99E-05
GO:0048522	positive regulation of cellular process	7.12E-05
GO:0019220	regulation of phosphate metabolic process	7.15E-05
GO:0051174	regulation of phosphorus metabolic process	7.15E-05
GO:2000026	regulation of multicellular organismal development	7.19E-05
GO:0044283	small molecule biosynthetic process	7.31E-05
GO:0035337	fatty-acyl-CoA metabolic process	7.52E-05
GO:0043542	endothelial cell migration	7.58E-05
GO:0016310	phosphorylation	7.94E-05
GO:0051128	regulation of cellular component organization	8.40E-05
GO:0019432	triglyceride biosynthetic process	8.48E-05
GO:0046504	glycerol ether biosynthetic process	8.51E-05
GO:0001932	regulation of protein phosphorylation	9.34E-05
GO:0009719	response to endogenous stimulus	9.42E-05
GO:0006091	generation of precursor metabolites and energy	9.90E-05
GO:0051271	negative regulation of cellular component movement	1.10E-04
GO:0006793	phosphorus metabolic process	1.12E-04
GO:0006796	phosphate-containing compound metabolic process	1.12E-04
GO:0010810	regulation of cell-substrate adhesion	1.19E-04
GO:0008354	germ cell migration	1.21E-04
GO:0009109	coenzyme catabolic process	1.21E-04
GO:0055085	transmembrane transport	1.22E-04
GO:0060749	mammary gland alveolus development	1.30E-04
GO:0061377	mammary gland lobule development	1.30E-04
GO:0001944	vasculature development	1.37E-04
GO:0009150	purine ribonucleotide metabolic process	1.37E-04
GO:0030336	negative regulation of cell migration	1.39E-04
GO:0007266	Rho protein signal transduction	1.40E-04
GO:0042493	response to drug	1.42E-04

GO:0016126	sterol biosynthetic process	1.49E-04
GO:0014070	response to organic cyclic compound	1.55E-04
GO:0042127	regulation of cell proliferation	1.58E-04
GO:0009141	nucleoside triphosphate metabolic process	1.63E-04
GO:0070887	cellular response to chemical stimulus	1.67E-04
GO:0043410	positive regulation of MAPK cascade	1.69E-04
GO:0051347	positive regulation of transferase activity	1.71E-04
GO:2000146	negative regulation of cell motility	1.74E-04
GO:0009199	ribonucleoside triphosphate metabolic process	1.77E-04
GO:0051188	cofactor biosynthetic process	1.85E-04
GO:0001936	regulation of endothelial cell proliferation	1.95E-04
GO:0034599	cellular response to oxidative stress	1.95E-04
GO:0072358	cardiovascular system development	1.99E-04
GO:0072359	circulatory system development	1.99E-04
GO:0051186	cofactor metabolic process	2.00E-04
GO:0009144	purine nucleoside triphosphate metabolic process	2.03E-04
GO:0038084	vascular endothelial growth factor signaling pathway	2.06E-04
GO:0008610	lipid biosynthetic process	2.20E-04
GO:0006464	cellular protein modification process	2.25E-04
GO:0036211	protein modification process	2.25E-04
GO:0006629	lipid metabolic process	2.27E-04
GO:0034655	nucleobase-containing compound catabolic process	2.52E-04
GO:0050673	epithelial cell proliferation	2.56E-04
GO:0006695	cholesterol biosynthetic process	2.60E-04
GO:0006195	purine nucleotide catabolic process	2.75E-04
GO:0045785	positive regulation of cell adhesion	3.00E-04
GO:0006468	protein phosphorylation	3.04E-04
GO:0009205	purine ribonucleoside triphosphate metabolic process	3.26E-04
GO:0042180	cellular ketone metabolic process	3.27E-04
GO:0044271	cellular nitrogen compound biosynthetic process	3.39E-04
GO:0072523	purine-containing compound catabolic process	3.41E-04
GO:0006066	alcohol metabolic process	3.50E-04
GO:0001525	angiogenesis	3.64E-04
GO:0001568	blood vessel development	3.66E-04

GO:0009725	response to hormone stimulus	3.79E-04
GO:0043085	positive regulation of catalytic activity	3.84E-04
GO:0007010	cytoskeleton organization	3.87E-04
GO:0043408	regulation of MAPK cascade	4.09E-04
GO:0033555	multicellular organismal response to stress	4.43E-04
GO:0043648	dicarboxylic acid metabolic process	4.43E-04
GO:0045445	myoblast differentiation	4.51E-04
GO:0051187	cofactor catabolic process	4.51E-04
GO:0048646	anatomical structure formation involved in morphogenesis	4.56E-04
GO:0033674	positive regulation of kinase activity	4.57E-04
GO:0007243	intracellular protein kinase cascade	4.62E-04
GO:0045859	regulation of protein kinase activity	4.64E-04
GO:0009165	nucleotide biosynthetic process	4.87E-04
GO:0019227	neuronal action potential propagation	5.00E-04
GO:0048518	positive regulation of biological process	5.00E-04
GO:0043412	macromolecule modification	5.02E-04
GO:0045595	regulation of cell differentiation	5.10E-04
GO:0007215	glutamate receptor signaling pathway	5.23E-04
GO:0071407	cellular response to organic cyclic compound	5.23E-04
GO:0006639	acylglycerol metabolic process	5.27E-04
GO:0009636	response to toxin	5.27E-04
GO:0007265	Ras protein signal transduction	5.30E-04
GO:0044267	cellular protein metabolic process	5.45E-04
GO:0006637	acyl-CoA metabolic process	5.46E-04
GO:0034637	cellular carbohydrate biosynthetic process	5.46E-04
GO:0035383	thioester metabolic process	5.46E-04
GO:0006638	neutral lipid metabolic process	5.84E-04
GO:0034654	nucleobase-containing compound biosynthetic process	5.97E-04
GO:0009166	nucleotide catabolic process	6.08E-04
GO:0065009	regulation of molecular function	6.10E-04
GO:0019752	carboxylic acid metabolic process	6.23E-04
GO:0043436	oxoacid metabolic process	6.23E-04
GO:0048513	organ development	6.28E-04
GO:0051349	positive regulation of lyase activity	6.29E-04

GO:0006082	organic acid metabolic process	6.40E-04
GO:0006112	energy reserve metabolic process	6.40E-04
GO:0006694	steroid biosynthetic process	6.46E-04
GO:0035295	tube development	6.55E-04
GO:0007155	cell adhesion	6.58E-04
GO:0022610	biological adhesion	6.58E-04
GO:0050905	neuromuscular process	6.78E-04
GO:0006641	triglyceride metabolic process	6.79E-04
GO:0046700	heterocycle catabolic process	6.82E-04
GO:0016125	sterol metabolic process	7.26E-04
GO:1900542	regulation of purine nucleotide metabolic process	7.26E-04
GO:0044270	cellular nitrogen compound catabolic process	7.36E-04
GO:0006821	chloride transport	8.24E-04
GO:0009146	purine nucleoside triphosphate catabolic process	8.37E-04
GO:0048523	negative regulation of cellular process	8.64E-04
GO:0006140	regulation of nucleotide metabolic process	8.87E-04
GO:0006836	neurotransmitter transport	9.31E-04
GO:0010469	regulation of receptor activity	9.42E-04
GO:0042981	regulation of apoptotic process	9.45E-04
GO:0009143	nucleoside triphosphate catabolic process	9.88E-04
GO:0006820	anion transport	9.90E-04
GO:0050678	regulation of epithelial cell proliferation	1.06E-03
GO:0015936	coenzyme A metabolic process	1.10E-03
GO:0007565	female pregnancy	1.10E-03
GO:0006915	apoptotic process	1.13E-03
GO:0051339	regulation of lyase activity	1.13E-03
GO:0030001	metal ion transport	1.15E-03
GO:0043067	regulation of programmed cell death	1.19E-03
GO:0008219	cell death	1.19E-03
GO:0010839	negative regulation of keratinocyte proliferation	1.22E-03
GO:0034063	stress granule assembly	1.22E-03
GO:0034614	cellular response to reactive oxygen species	1.22E-03
GO:0070120	ciliary neurotrophic factor-mediated signaling pathway	1.22E-03
GO:0008202	steroid metabolic process	1.24E-03

GO:0016265	death	1.24E-03
GO:0035924	cellular response to vascular endothelial growth factor stimulus	1.25E-03
GO:0008285	negative regulation of cell proliferation	1.25E-03
GO:0008088	axon cargo transport	1.25E-03
GO:0031399	regulation of protein modification process	1.27E-03
GO:0048010	vascular endothelial growth factor receptor signaling pathway	1.29E-03
GO:0050790	regulation of catalytic activity	1.31E-03
GO:0071363	cellular response to growth factor stimulus	1.32E-03
GO:0051056	regulation of small GTPase mediated signal transduction	1.32E-03
GO:0006662	glycerol ether metabolic process	1.34E-03
GO:0045860	positive regulation of protein kinase activity	1.39E-03
GO:0048732	gland development	1.40E-03
GO:0030073	insulin secretion	1.42E-03
GO:0042692	muscle cell differentiation	1.47E-03
GO:0012501	programmed cell death	1.47E-03
GO:0050900	leukocyte migration	1.47E-03
GO:0009203	ribonucleoside triphosphate catabolic process	1.49E-03
GO:0009207	purine ribonucleoside triphosphate catabolic process	1.49E-03
GO:0051649	establishment of localization in cell	1.50E-03
GO:0007160	cell-matrix adhesion	1.51E-03
GO:0016051	carbohydrate biosynthetic process	1.52E-03
GO:0046903	secretion	1.53E-03
GO:0051146	striated muscle cell differentiation	1.53E-03
GO:0010941	regulation of cell death	1.63E-03
GO:0007611	learning or memory	1.63E-03
GO:0034765	regulation of ion transmembrane transport	1.63E-03
GO:0007190	activation of adenylate cyclase activity	1.64E-03
GO:0015807	L-amino acid transport	1.64E-03
GO:0060736	prostate gland growth	1.65E-03
GO:0050890	cognition	1.65E-03
GO:0071780	mitotic cell cycle G2-M transition checkpoint	1.65E-03
GO:0070848	response to growth factor stimulus	1.71E-03
GO:0010975	regulation of neuron projection development	1.72E-03
GO:0018130	heterocycle biosynthetic process	1.72E-03

GO:0032412	regulation of ion transmembrane transporter activity	1.72E-03
GO:0043434	response to peptide hormone stimulus	1.74E-03
GO:0001894	tissue homeostasis	1.81E-03
GO:0051641	cellular localization	1.83E-03
GO:0048519	negative regulation of biological process	1.87E-03
GO:0006184	GTP catabolic process	1.89E-03
GO:0009154	purine ribonucleotide catabolic process	1.89E-03
GO:0044057	regulation of system process	1.89E-03
GO:0003001	generation of a signal involved in cell-cell signaling	1.99E-03
GO:0023061	signal release	1.99E-03
GO:0045762	positive regulation of adenylate cyclase activity	2.02E-03
GO:0009261	ribonucleotide catabolic process	2.04E-03
GO:0044093	positive regulation of molecular function	2.11E-03
GO:0030072	peptide hormone secretion	2.15E-03
GO:0007214	gamma-aminobutyric acid signaling pathway	2.16E-03
GO:1900449	regulation of glutamate receptor signaling pathway	2.16E-03
GO:0018904	organic ether metabolic process	2.17E-03
GO:0055002	striated muscle cell development	2.17E-03
GO:0045666	positive regulation of neuron differentiation	2.19E-03
GO:0015810	aspartate transport	2.34E-03
GO:0045761	regulation of adenylate cyclase activity	2.35E-03
GO:0046364	monosaccharide biosynthetic process	2.35E-03
GO:0043129	surfactant homeostasis	2.38E-03
GO:0048875	chemical homeostasis within a tissue	2.38E-03
GO:0043066	negative regulation of apoptotic process	2.40E-03
GO:0031281	positive regulation of cyclase activity	2.46E-03
GO:0046165	alcohol biosynthetic process	2.58E-03
GO:0006423	cysteinyl-tRNA aminoacylation	2.59E-03
GO:0010868	negative regulation of triglyceride biosynthetic process	2.59E-03
GO:0035617	stress granule disassembly	2.59E-03
GO:0042231	interleukin-13 biosynthetic process	2.59E-03
GO:0015837	amine transport	2.62E-03
GO:0045664	regulation of neuron differentiation	2.68E-03
GO:0007018	microtubule-based movement	2.73E-03

GO:0010039	response to iron ion	2.76E-03
GO:0050926	regulation of positive chemotaxis	2.76E-03
GO:0072384	organelle transport along microtubule	2.76E-03
GO:0043069	negative regulation of programmed cell death	2.84E-03
GO:0022898	regulation of transmembrane transporter activity	2.86E-03
GO:0001569	patterning of blood vessels	2.97E-03
GO:0002790	peptide secretion	2.97E-03
GO:0008038	neuron recognition	2.97E-03
GO:0046039	GTP metabolic process	3.01E-03
GO:0006950	response to stress	3.03E-03
GO:0031344	regulation of cell projection organization	3.07E-03
GO:0032268	regulation of cellular protein metabolic process	3.07E-03
GO:0031279	regulation of cyclase activity	3.08E-03
GO:0044255	cellular lipid metabolic process	3.10E-03
GO:0033002	muscle cell proliferation	3.14E-03
GO:0048638	regulation of developmental growth	3.14E-03
GO:0009991	response to extracellular stimulus	3.15E-03
GO:0008203	cholesterol metabolic process	3.31E-03
GO:0055001	muscle cell development	3.39E-03
GO:0015698	inorganic anion transport	3.42E-03
GO:0044262	cellular carbohydrate metabolic process	3.42E-03
GO:0060548	negative regulation of cell death	3.44E-03
GO:0051592	response to calcium ion	3.46E-03
GO:0042596	fear response	3.48E-03
GO:0035023	regulation of Rho protein signal transduction	3.49E-03
GO:0010594	regulation of endothelial cell migration	3.53E-03
GO:0000302	response to reactive oxygen species	3.58E-03
GO:0055114	oxidation-reduction process	3.67E-03
GO:0008015	blood circulation	3.72E-03
GO:0006006	glucose metabolic process	3.84E-03
GO:0003013	circulatory system process	3.89E-03
GO:0006897	endocytosis	3.92E-03
GO:0009743	response to carbohydrate stimulus	3.93E-03
GO:0006101	citrate metabolic process	3.94E-03

GO:0006108	malate metabolic process	3.94E-03
GO:0006208	pyrimidine base catabolic process	3.94E-03
GO:0006538	glutamate catabolic process	3.94E-03
GO:0048268	clathrin coat assembly	3.94E-03
GO:0051775	response to redox state	3.94E-03
GO:0051938	L-glutamate import	3.94E-03
GO:0072350	tricarboxylic acid metabolic process	3.94E-03
GO:0071844	cellular component assembly at cellular level	3.95E-03
GO:0009914	hormone transport	4.06E-03
GO:0019318	hexose metabolic process	4.08E-03
GO:0040017	positive regulation of locomotion	4.29E-03
GO:0072522	purine-containing compound biosynthetic process	4.29E-03
GO:0008299	isoprenoid biosynthetic process	4.32E-03
GO:0001937	negative regulation of endothelial cell proliferation	4.32E-03
GO:0006835	dicarboxylic acid transport	4.43E-03
GO:0006979	response to oxidative stress	4.47E-03
GO:0009123	nucleoside monophosphate metabolic process	4.49E-03
GO:0030816	positive regulation of cAMP metabolic process	4.49E-03
GO:0030819	positive regulation of cAMP biosynthetic process	4.49E-03
GO:0045333	cellular respiration	4.49E-03
GO:0046620	regulation of organ growth	4.49E-03
GO:0051272	positive regulation of cellular component movement	4.53E-03
GO:0009056	catabolic process	4.54E-03
GO:0008016	regulation of heart contraction	4.55E-03
GO:0006164	purine nucleotide biosynthetic process	4.56E-03
GO:0008284	positive regulation of cell proliferation	4.56E-03
GO:0070507	regulation of microtubule cytoskeleton organization	4.57E-03
GO:0050796	regulation of insulin secretion	4.88E-03
GO:0022603	regulation of anatomical structure morphogenesis	4.90E-03
GO:0042058	regulation of epidermal growth factor receptor signaling pathway	4.90E-03
GO:0070301	cellular response to hydrogen peroxide	4.90E-03
GO:0032886	regulation of microtubule-based process	4.97E-03
GO:0048167	regulation of synaptic plasticity	5.03E-03
GO:0007044	cell-substrate junction assembly	5.06E-03

GO:0048871	multicellular organismal homeostasis	5.13E-03
GO:0006812	cation transport	5.15E-03
GO:0009110	vitamin biosynthetic process	5.29E-03
GO:0005975	carbohydrate metabolic process	5.30E-03
GO:0030335	positive regulation of cell migration	5.38E-03
GO:0050767	regulation of neurogenesis	5.45E-03
GO:0071310	cellular response to organic substance	5.47E-03
GO:0030817	regulation of cAMP biosynthetic process	5.52E-03
GO:0071702	organic substance transport	5.57E-03
GO:0046847	filopodium assembly	5.61E-03
GO:0001964	startle response	5.80E-03
GO:0007413	axonal fasciculation	5.80E-03
GO:0071312	cellular response to alkaloid	5.80E-03
GO:0042364	water-soluble vitamin biosynthetic process	6.08E-03
GO:0042415	norepinephrine metabolic process	6.08E-03
GO:0046113	nucleobase catabolic process	6.08E-03
GO:0048514	blood vessel morphogenesis	6.27E-03
GO:0046879	hormone secretion	6.32E-03
GO:2000147	positive regulation of cell motility	6.32E-03
GO:0031532	actin cytoskeleton reorganization	6.38E-03
GO:0015833	peptide transport	6.39E-03
GO:0031111	negative regulation of microtubule polymerization or depolymerization	6.40E-03
GO:0009611	response to wounding	6.52E-03
GO:0048771	tissue remodeling	6.56E-03
GO:0010647	positive regulation of cell communication	6.60E-03
GO:0030814	regulation of cAMP metabolic process	6.63E-03
GO:0043269	regulation of ion transport	6.66E-03
GO:0044248	cellular catabolic process	6.70E-03
GO:0007618	mating	6.77E-03
GO:0034103	regulation of tissue remodeling	6.77E-03
GO:0030804	positive regulation of cyclic nucleotide biosynthetic process	7.09E-03
GO:0010811	positive regulation of cell-substrate adhesion	7.23E-03
GO:0019319	hexose biosynthetic process	7.32E-03
GO:0060284	regulation of cell development	7.37E-03

GO:0006106	fumarate metabolic process	7.38E-03
GO:0009448	gamma-aminobutyric acid metabolic process	7.38E-03
GO:0010881	regulation of cardiac muscle contraction by regulation of the release of sequestered calcium ion	7.38E-03
GO:0019896	axon transport of mitochondrion	7.38E-03
GO:0042816	vitamin B6 metabolic process	7.38E-03
GO:0042819	vitamin B6 biosynthetic process	7.38E-03
GO:0043490	malate-aspartate shuttle	7.38E-03
GO:0048014	Tie receptor signaling pathway	7.38E-03
GO:0060312	regulation of blood vessel remodeling	7.38E-03
GO:0070777	D-aspartate transport	7.38E-03
GO:0070779	D-aspartate import	7.38E-03
GO:0019538	protein metabolic process	7.42E-03
GO:0030260	entry into host cell	7.43E-03
GO:0030949	positive regulation of vascular endothelial growth factor receptor signaling pathway	7.43E-03
GO:0044409	entry into host	7.43E-03
GO:0051806	entry into cell of other organism involved in symbiotic interaction	7.43E-03
GO:0051828	entry into other organism involved in symbiotic interaction	7.43E-03
GO:0052126	movement in host environment	7.43E-03
GO:0052192	movement in environment of other organism involved in symbiotic interaction	7.43E-03
GO:0048585	negative regulation of response to stimulus	7.56E-03
GO:0033865	nucleoside bisphosphate metabolic process	7.67E-03
GO:0023056	positive regulation of signaling	7.77E-03
GO:0009066	aspartate family amino acid metabolic process	7.83E-03
GO:0071495	cellular response to endogenous stimulus	7.86E-03
GO:0007017	microtubule-based process	7.86E-03
GO:0031667	response to nutrient levels	7.86E-03
GO:0030810	positive regulation of nucleotide biosynthetic process	7.89E-03
GO:0044264	cellular polysaccharide metabolic process	7.89E-03
GO:1900373	positive regulation of purine nucleotide biosynthetic process	7.89E-03
GO:0008286	insulin receptor signaling pathway	8.06E-03
GO:0050804	regulation of synaptic transmission	8.06E-03
GO:0001505	regulation of neurotransmitter levels	8.07E-03
GO:0001655	urogenital system development	8.10E-03
GO:0006936	muscle contraction	8.10E-03

GO:0032409	regulation of transporter activity	8.15E-03
GO:0070588	calcium ion transmembrane transport	8.16E-03
GO:0010740	positive regulation of intracellular protein kinase cascade	8.17E-03
GO:0009888	tissue development	8.47E-03
GO:0010648	negative regulation of cell communication	8.72E-03
GO:0023057	negative regulation of signaling	8.72E-03
GO:0030801	positive regulation of cyclic nucleotide metabolic process	8.76E-03
GO:0035335	peptidyl-tyrosine dephosphorylation	8.76E-03
GO:0007171	activation of transmembrane receptor protein tyrosine kinase activity	8.77E-03
GO:0032331	negative regulation of chondrocyte differentiation	8.77E-03
GO:0045749	negative regulation of S phase of mitotic cell cycle	8.77E-03
GO:0060292	long term synaptic depression	8.77E-03
GO:0060384	innervation	8.77E-03
GO:0008584	male gonad development	8.81E-03
GO:0010906	regulation of glucose metabolic process	8.84E-03
GO:0035265	organ growth	8.84E-03
GO:0090276	regulation of peptide hormone secretion	8.90E-03
GO:0051130	positive regulation of cellular component organization	8.94E-03
GO:0090287	regulation of cellular response to growth factor stimulus	9.11E-03
GO:0060443	mammary gland morphogenesis	9.18E-03
GO:0030879	mammary gland development	9.23E-03
GO:0032940	secretion by cell	9.29E-03
GO:0015813	L-glutamate transport	9.33E-03
GO:0031113	regulation of microtubule polymerization	9.33E-03
GO:0043279	response to alkaloid	9.33E-03
GO:0046546	development of primary male sexual characteristics	9.45E-03
GO:0050680	negative regulation of epithelial cell proliferation	9.68E-03
GO:0014065	phosphatidylinositol 3-kinase cascade	9.70E-03
GO:0044237	cellular metabolic process	9.81E-03

C

Accession	GO Term Biological Processes enriched in down regulated genes	Robin
GO:0030030	cell projection organization	1.20E-07
GO:0016043	cellular component organization	1.08E-06
GO:0071840	cellular component organization or biogenesis	2.18E-06
GO:0071842	cellular component organization at cellular level	2.63E-06
GO:0071841	cellular component organization or biogenesis at cellular level	4.78E-06
GO:0009653	anatomical structure morphogenesis	5.18E-06
GO:0048858	cell projection morphogenesis	6.27E-06
GO:0032990	cell part morphogenesis	8.10E-06
GO:0048869	cellular developmental process	1.15E-05
GO:0048667	cell morphogenesis involved in neuron differentiation	1.50E-05
GO:0000904	cell morphogenesis involved in differentiation	2.92E-05
GO:0000902	cell morphogenesis	3.21E-05
GO:0030154	cell differentiation	4.89E-05
GO:0032502	developmental process	5.12E-05
GO:0048812	neuron projection morphogenesis	5.21E-05
GO:0023052	signaling	5.49E-05
GO:0048856	anatomical structure development	7.29E-05
GO:0007399	nervous system development	8.05E-05
GO:0032989	cellular component morphogenesis	8.25E-05
GO:0007154	cell communication	8.67E-05
GO:0007165	signal transduction	9.39E-05
GO:0016568	chromatin modification	1.33E-04
GO:0040011	locomotion	1.95E-04
GO:0016570	histone modification	1.98E-04

GO:0007275	multicellular organismal development	1.98E-04
GO:0042522	regulation of tyrosine phosphorylation of Stat5 protein	1.99E-04
GO:0006928	cellular component movement	2.00E-04
GO:0031175	neuron projection development	2.03E-04
GO:0043954	cellular component maintenance	2.12E-04
GO:0048666	neuron development	2.22E-04
GO:0016569	covalent chromatin modification	2.49E-04
GO:0007409	axonogenesis	3.46E-04
GO:0042506	tyrosine phosphorylation of Stat5 protein	3.61E-04
GO:0007411	axon guidance	3.91E-04
GO:0050794	regulation of cellular process	4.05E-04
GO:0009790	embryo development	4.22E-04
GO:0048731	system development	4.66E-04
GO:0007167	enzyme linked receptor protein signaling pathway	5.18E-04
GO:0032501	multicellular organismal process	5.98E-04
GO:0045494	photoreceptor cell maintenance	6.02E-04
GO:0007169	transmembrane receptor protein tyrosine kinase signaling pathway	6.18E-04
GO:0048699	generation of neurons	7.18E-04
GO:0035234	germ cell programmed cell death	7.41E-04
GO:0051716	cellular response to stimulus	7.82E-04
GO:0022008	neurogenesis	8.44E-04
GO:0030182	neuron differentiation	9.80E-04
GO:0034204	lipid translocation	1.00E-03
GO:0045332	phospholipid translocation	1.00E-03
GO:0007018	microtubule-based movement	1.16E-03
GO:0002028	regulation of sodium ion transport	1.39E-03
GO:0022029	telencephalon cell migration	1.39E-03
GO:0022610	biological adhesion	1.40E-03
GO:0007155	cell adhesion	1.40E-03
GO:0065007	biological regulation	1.42E-03
GO:0033152	immunoglobulin V(D)J recombination	1.47E-03
GO:0048496	maintenance of organ identity	1.47E-03
GO:0006325	chromatin organization	1.51E-03
GO:0018205	peptidyl-lysine modification	1.54E-03

GO:0007166	cell surface receptor signaling pathway	1.55E-03
GO:0007420	brain development	1.57E-03
GO:0060113	inner ear receptor cell differentiation	1.57E-03
GO:0021885	forebrain cell migration	1.76E-03
GO:0042523	positive regulation of tyrosine phosphorylation of Stat5 protein	1.90E-03
GO:0006357	regulation of transcription from RNA polymerase II promoter	2.06E-03
GO:0050793	regulation of developmental process	2.18E-03
GO:0010390	histone monoubiquitination	2.28E-03
GO:0090286	cytoskeletal anchoring at nuclear membrane	2.43E-03
GO:0002024	diet induced thermogenesis	2.43E-03
GO:0048468	cell development	2.44E-03
GO:0042490	mechanoreceptor differentiation	2.44E-03
GO:0009987	cellular process	2.53E-03
GO:0051270	regulation of cellular component movement	2.59E-03
GO:0097035	regulation of membrane lipid distribution	2.70E-03
GO:0007517	muscle organ development	2.85E-03
GO:0048513	organ development	2.87E-03
GO:0046209	nitric oxide metabolic process	2.99E-03
GO:0009628	response to abiotic stimulus	3.13E-03
GO:0050789	regulation of biological process	3.14E-03
GO:0048839	inner ear development	3.47E-03
GO:0060425	lung morphogenesis	3.61E-03
GO:0060501	positive regulation of epithelial cell proliferation involved in lung morphogenesis	3.61E-03
GO:0060013	righting reflex	3.61E-03
GO:0060429	epithelium development	3.65E-03
GO:0008045	motor axon guidance	3.68E-03
GO:0044253	positive regulation of multicellular organismal metabolic process	3.68E-03
GO:0007417	central nervous system development	3.78E-03
GO:0007266	Rho protein signal transduction	3.85E-03
GO:0048870	cell motility	3.86E-03
GO:0051674	localization of cell	3.86E-03
GO:0051173	positive regulation of nitrogen compound metabolic process	3.86E-03
GO:0001539	ciliary or flagellar motility	4.25E-03
GO:0035272	exocrine system development	4.31E-03

GO:0060541	respiratory system development	4.52E-03
GO:0061061	muscle structure development	4.60E-03
GO:0016925	protein sumoylation	4.86E-03
GO:0018193	peptidyl-amino acid modification	4.95E-03
GO:0050957	equilibrioception	4.99E-03
GO:0035518	histone H2A monoubiquitination	4.99E-03
GO:0055091	phospholipid homeostasis	4.99E-03
GO:0033235	positive regulation of protein sumoylation	4.99E-03
GO:2000794	regulation of epithelial cell proliferation involved in lung morphogenesis	4.99E-03
GO:0055098	response to low-density lipoprotein particle stimulus	4.99E-03
GO:0022028	tangential migration from the subventricular zone to the olfactory bulb	4.99E-03
GO:0043967	histone H4 acetylation	5.10E-03
GO:0045935	positive regulation of nucleobase-containing compound metabolic process	5.36E-03
GO:0051276	chromosome organization	5.41E-03
GO:0021772	olfactory bulb development	5.53E-03
GO:0021988	olfactory lobe development	5.53E-03
GO:0051050	positive regulation of transport	5.82E-03
GO:0032879	regulation of localization	5.97E-03
GO:0009888	tissue development	6.14E-03
GO:0002790	peptide secretion	6.21E-03
GO:0009268	response to pH	6.24E-03
GO:0051897	positive regulation of protein kinase B signaling cascade	6.46E-03
GO:0060502	epithelial cell proliferation involved in lung morphogenesis	6.59E-03
GO:0021537	telencephalon development	6.74E-03
GO:0030324	lung development	7.00E-03
GO:0044246	regulation of multicellular organismal metabolic process	7.01E-03
GO:0016573	histone acetylation	7.15E-03
GO:0032388	positive regulation of intracellular transport	7.15E-03
GO:0031344	regulation of cell projection organization	7.24E-03
GO:0010975	regulation of neuron projection development	7.34E-03
GO:0031328	positive regulation of cellular biosynthetic process	7.52E-03
GO:0006897	endocytosis	7.53E-03
GO:0040012	regulation of locomotion	7.63E-03
GO:0051179	localization	7.80E-03

GO:0018393	internal peptidyl-lysine acetylation	7.81E-03
GO:0030323	respiratory tube development	7.83E-03
GO:0042509	regulation of tyrosine phosphorylation of STAT protein	8.04E-03
GO:0048266	behavioral response to pain	8.39E-03
GO:0031958	corticosteroid receptor signaling pathway	8.39E-03
GO:0042921	glucocorticoid receptor signaling pathway	8.39E-03
GO:0043949	regulation of cAMP-mediated signaling	8.39E-03
GO:0033233	regulation of protein sumoylation	8.39E-03
GO:0009887	organ morphogenesis	8.46E-03
GO:0010927	cellular component assembly involved in morphogenesis	8.51E-03
GO:0018394	peptidyl-lysine acetylation	8.51E-03
GO:0050954	sensory perception of mechanical stimulus	8.51E-03
GO:0006513	protein monoubiquitination	8.71E-03
GO:0072332	signal transduction by p53 class mediator resulting in induction of apoptosis	8.71E-03
GO:0006935	chemotaxis	9.10E-03
GO:0042330	taxis	9.10E-03
GO:0009891	positive regulation of biosynthetic process	9.36E-03
GO:0043583	ear development	9.38E-03
GO:0015833	peptide transport	9.46E-03
GO:0006475	internal protein amino acid acetylation	9.64E-03
GO:0048646	anatomical structure formation involved in morphogenesis	9.65E-03
GO:0060038	cardiac muscle cell proliferation	9.65E-03
GO:0016574	histone ubiquitination	9.65E-03
GO:0009605	response to external stimulus	9.76E-03

D

Accession	GO Term Biological Processes enriched in down regulated genes	Canary
GO:0071842	cellular component organization at cellular level	1.18E-14
GO:0016043	cellular component organization	1.46E-14
GO:0071841	cellular component organization or biogenesis at cellular level	5.97E-14
GO:0006996	organelle organization	8.99E-14
GO:0071840	cellular component organization or biogenesis	2.71E-13
GO:0051276	chromosome organization	3.27E-10
GO:0016568	chromatin modification	9.74E-10
GO:0009987	cellular process	1.55E-09
GO:0022402	cell cycle process	2.94E-09
GO:0055085	transmembrane transport	1.51E-08
GO:0006281	DNA repair	6.32E-08
GO:0007010	cytoskeleton organization	9.71E-08
GO:0007049	cell cycle	1.48E-07
GO:0022403	cell cycle phase	4.48E-07
GO:0016570	histone modification	9.72E-07
GO:0000278	mitotic cell cycle	1.24E-06
GO:0030030	cell projection organization	1.56E-06
GO:0042384	cilium assembly	1.93E-06
GO:0016569	covalent chromatin modification	2.10E-06
GO:0030031	cell projection assembly	2.64E-06
GO:0006325	chromatin organization	2.65E-06
GO:0051179	localization	2.68E-06
GO:0071897	DNA biosynthetic process	2.99E-06
GO:0007017	microtubule-based process	4.03E-06
GO:0051297	centrosome organization	4.80E-06
GO:0006260	DNA replication	4.94E-06
GO:0006811	ion transport	5.08E-06
GO:0007163	establishment or maintenance of cell polarity	7.90E-06
GO:0010927	cellular component assembly involved in morphogenesis	1.04E-05
GO:0006261	DNA-dependent DNA replication	1.07E-05

GO:0032502	developmental process	1.08E-05
GO:0006302	double-strand break repair	1.08E-05
GO:0060271	cilium morphogenesis	1.58E-05
GO:0018205	peptidyl-lysine modification	1.70E-05
GO:0032989	cellular component morphogenesis	1.73E-05
GO:0031023	microtubule organizing center organization	1.86E-05
GO:0032508	DNA duplex unwinding	2.19E-05
GO:0051298	centrosome duplication	2.19E-05
GO:0048869	cellular developmental process	2.66E-05
GO:0000902	cell morphogenesis	2.72E-05
GO:0006259	DNA metabolic process	3.07E-05
GO:0032392	DNA geometric change	3.35E-05
GO:0032501	multicellular organismal process	3.73E-05
GO:0000279	M phase	5.18E-05
GO:0048858	cell projection morphogenesis	5.27E-05
GO:0048856	anatomical structure development	5.29E-05
GO:0030010	establishment of cell polarity	5.62E-05
GO:0000226	microtubule cytoskeleton organization	5.67E-05
GO:0018394	peptidyl-lysine acetylation	8.04E-05
GO:0032990	cell part morphogenesis	9.36E-05
GO:0007098	centrosome cycle	9.39E-05
GO:0000724	double-strand break repair via homologous recombination	9.68E-05
GO:0051056	regulation of small GTPase mediated signal transduction	1.01E-04
GO:0016573	histone acetylation	1.10E-04
GO:0009653	anatomical structure morphogenesis	1.21E-04
GO:0000725	recombinational repair	1.25E-04
GO:0034968	histone lysine methylation	1.26E-04
GO:0006475	internal protein amino acid acetylation	1.30E-04
GO:0051647	nucleus localization	1.44E-04
GO:0018393	internal peptidyl-lysine acetylation	1.52E-04
GO:0000086	G2-M transition of mitotic cell cycle	1.58E-04
GO:0006464	cellular protein modification process	1.70E-04
GO:0036211	protein modification process	1.70E-04
GO:0061061	muscle structure development	1.72E-04

GO:0006310	DNA recombination	1.78E-04
GO:0050793	regulation of developmental process	1.93E-04
GO:0071702	organic substance transport	1.93E-04
GO:0006473	protein acetylation	2.03E-04
GO:0000132	establishment of mitotic spindle orientation	2.12E-04
GO:0010824	regulation of centrosome duplication	2.12E-04
GO:0031060	regulation of histone methylation	2.12E-04
GO:0051294	establishment of spindle orientation	2.12E-04
GO:0035023	regulation of Rho protein signal transduction	2.13E-04
GO:0043967	histone H4 acetylation	2.21E-04
GO:0051293	establishment of spindle localization	2.44E-04
GO:0051653	spindle localization	2.44E-04
GO:0022610	biological adhesion	2.76E-04
GO:0007155	cell adhesion	2.76E-04
GO:0000731	DNA synthesis involved in DNA repair	2.86E-04
GO:0031061	negative regulation of histone methylation	3.13E-04
GO:0051325	interphase	3.17E-04
GO:0016571	histone methylation	3.25E-04
GO:0046578	regulation of Ras protein signal transduction	3.54E-04
GO:0034661	ncRNA catabolic process	3.83E-04
GO:0046605	regulation of centrosome cycle	3.96E-04
GO:0048468	cell development	4.22E-04
GO:0030029	actin filament-based process	4.45E-04
GO:0007517	muscle organ development	4.63E-04
GO:0007266	Rho protein signal transduction	4.64E-04
GO:0006974	response to DNA damage stimulus	4.82E-04
GO:0043412	macromolecule modification	5.04E-04
GO:0051329	interphase of mitotic cell cycle	5.05E-04
GO:0043543	protein acylation	5.09E-04
GO:0000768	syncytium formation by plasma membrane fusion	5.70E-04
GO:0006301	postreplication repair	5.70E-04
GO:0051234	establishment of localization	5.95E-04
GO:0060537	muscle tissue development	5.96E-04
GO:0040001	establishment of mitotic spindle localization	6.48E-04

GO:0090286	cytoskeletal anchoring at nuclear membrane	7.16E-04
GO:0016075	rRNA catabolic process	7.56E-04
GO:0043966	histone H3 acetylation	8.17E-04
GO:0018193	peptidyl-amino acid modification	8.28E-04
GO:0043984	histone H4-K16 acetylation	9.29E-04
GO:0021955	central nervous system neuron axonogenesis	1.04E-03
GO:0006869	lipid transport	1.10E-03
GO:0007275	multicellular organismal development	1.12E-03
GO:0046777	protein autophosphorylation	1.12E-03
GO:0051960	regulation of nervous system development	1.14E-03
GO:0015695	organic cation transport	1.16E-03
GO:0051052	regulation of DNA metabolic process	1.16E-03
GO:0006479	protein methylation	1.18E-03
GO:0008213	protein alkylation	1.18E-03
GO:0000723	telomere maintenance	1.34E-03
GO:0030198	extracellular matrix organization	1.34E-03
GO:0043062	extracellular structure organization	1.34E-03
GO:0006928	cellular component movement	1.35E-03
GO:0006200	ATP catabolic process	1.38E-03
GO:0021834	chemorepulsion involved in embryonic olfactory bulb interneuron precursor migration	1.43E-03
GO:0030951	establishment or maintenance of microtubule cytoskeleton polarity	1.43E-03
GO:0034653	retinoic acid catabolic process	1.43E-03
GO:0050955	thermoception	1.43E-03
GO:0071321	cellular response to cGMP	1.43E-03
GO:0014706	striated muscle tissue development	1.52E-03
GO:0022029	telencephalon cell migration	1.54E-03
GO:0006622	protein targeting to lysosome	1.55E-03
GO:0006623	protein targeting to vacuole	1.55E-03
GO:0031062	positive regulation of histone methylation	1.55E-03
GO:0040023	establishment of nucleus localization	1.55E-03
GO:0051569	regulation of histone H3-K4 methylation	1.55E-03
GO:0072665	protein localization to vacuole	1.55E-03
GO:0072666	establishment of protein localization to vacuole	1.55E-03
GO:0032200	telomere organization	1.60E-03

GO:0006814	sodium ion transport	1.72E-03
GO:0033554	cellular response to stress	1.72E-03
GO:0060249	anatomical structure homeostasis	1.72E-03
GO:0034204	lipid translocation	1.73E-03
GO:0045332	phospholipid translocation	1.73E-03
GO:0021952	central nervous system projection neuron axonogenesis	1.73E-03
GO:0007044	cell-substrate junction assembly	1.75E-03
GO:0010876	lipid localization	1.81E-03
GO:0001843	neural tube closure	1.85E-03
GO:0022607	cellular component assembly	1.87E-03
GO:0007140	male meiosis	1.92E-03
GO:0051568	histone H3-K4 methylation	1.92E-03
GO:0033043	regulation of organelle organization	1.96E-03
GO:0030859	polarized epithelial cell differentiation	2.00E-03
GO:0051571	positive regulation of histone H3-K4 methylation	2.00E-03
GO:0070486	leukocyte aggregation	2.00E-03
GO:0007059	chromosome segregation	2.12E-03
GO:0065007	biological regulation	2.20E-03
GO:0060606	tube closure	2.21E-03
GO:0007127	meiosis I	2.26E-03
GO:0051716	cellular response to stimulus	2.35E-03
GO:0097035	regulation of membrane lipid distribution	2.36E-03
GO:0051726	regulation of cell cycle	2.40E-03
GO:0030154	cell differentiation	2.43E-03
GO:0007126	meiosis	2.46E-03
GO:0051327	M phase of meiotic cell cycle	2.46E-03
GO:0051640	organelle localization	2.46E-03
GO:0021885	forebrain cell migration	2.61E-03
GO:0001556	oocyte maturation	2.74E-03
GO:0006949	syncytium formation	2.74E-03
GO:0007099	centriole replication	2.74E-03
GO:0050919	negative chemotaxis	2.74E-03
GO:0000280	nuclear division	2.78E-03
GO:0007067	mitosis	2.78E-03

GO:0007520	myoblast fusion	2.80E-03
GO:0048666	neuron development	2.81E-03
GO:0007265	Ras protein signal transduction	2.93E-03
GO:0022604	regulation of cell morphogenesis	3.03E-03
GO:0051321	meiotic cell cycle	3.03E-03
GO:0014020	primary neural tube formation	3.11E-03
GO:0006468	protein phosphorylation	3.21E-03
GO:0065008	regulation of biological quality	3.22E-03
GO:0006810	transport	3.26E-03
GO:0015914	phospholipid transport	3.33E-03
GO:0000087	M phase of mitotic cell cycle	3.50E-03
GO:0055088	lipid homeostasis	3.57E-03
GO:0017158	regulation of calcium ion-dependent exocytosis	3.59E-03
GO:0061180	mammary gland epithelium development	3.67E-03
GO:0008015	blood circulation	4.09E-03
GO:0043981	histone H4-K5 acetylation	4.13E-03
GO:0043982	histone H4-K8 acetylation	4.13E-03
GO:0022028	tangential migration from the subventricular zone to the olfactory bulb	4.25E-03
GO:0009111	vitamin catabolic process	4.25E-03
GO:0042363	fat-soluble vitamin catabolic process	4.25E-03
GO:0043983	histone H4-K12 acetylation	4.25E-03
GO:0048096	chromatin-mediated maintenance of transcription	4.25E-03
GO:0051570	regulation of histone H3-K9 methylation	4.25E-03
GO:0060177	regulation of angiotensin metabolic process	4.25E-03
GO:0003013	circulatory system process	4.38E-03
GO:0006820	anion transport	4.57E-03
GO:0007143	female meiosis	4.66E-03
GO:0048841	regulation of axon extension involved in axon guidance	4.66E-03
GO:0050667	homocysteine metabolic process	4.66E-03
GO:0007160	cell-matrix adhesion	4.79E-03
GO:0008037	cell recognition	4.91E-03
GO:0031175	neuron projection development	4.93E-03
GO:0007399	nervous system development	5.07E-03
GO:0033152	immunoglobulin V(D)J recombination	5.08E-03

GO:0000019	regulation of mitotic recombination	5.08E-03
GO:0001778	plasma membrane repair	5.08E-03
GO:0010633	negative regulation of epithelial cell migration	5.08E-03
GO:0017014	protein nitrosylation	5.08E-03
GO:0018076	N-terminal peptidyl-lysine acetylation	5.08E-03
GO:0018119	peptidyl-cysteine S-nitrosylation	5.08E-03
GO:0019985	translesion synthesis	5.08E-03
GO:0021831	embryonic olfactory bulb interneuron precursor migration	5.08E-03
GO:0030301	cholesterol transport	5.08E-03
GO:0030952	establishment or maintenance of cytoskeleton polarity	5.08E-03
GO:0034983	peptidyl-lysine deacetylation	5.08E-03
GO:0044268	multicellular organismal protein metabolic process	5.08E-03
GO:0051573	negative regulation of histone H3-K9 methylation	5.08E-03
GO:0070305	response to cGMP	5.08E-03
GO:0070932	histone H3 deacetylation	5.08E-03
GO:0071670	smooth muscle cell chemotaxis	5.08E-03
GO:0006312	mitotic recombination	5.23E-03
GO:0031929	TOR signaling cascade	5.23E-03
GO:0022603	regulation of anatomical structure morphogenesis	5.25E-03
GO:0006812	cation transport	5.38E-03
GO:0060284	regulation of cell development	5.62E-03
GO:0006282	regulation of DNA repair	5.66E-03
GO:0048513	organ development	5.75E-03
GO:0001738	morphogenesis of a polarized epithelium	5.79E-03
GO:0017156	calcium ion-dependent exocytosis	5.90E-03
GO:0015918	sterol transport	5.96E-03
GO:0003081	regulation of systemic arterial blood pressure by renin-angiotensin	6.00E-03
GO:0070207	protein homotrimerization	6.00E-03
GO:0030036	actin cytoskeleton organization	6.17E-03
GO:0010564	regulation of cell cycle process	6.20E-03
GO:0006865	amino acid transport	6.25E-03
GO:0046942	carboxylic acid transport	6.54E-03
GO:0048285	organelle fission	6.56E-03
GO:0051656	establishment of organelle localization	6.70E-03

GO:0006801	superoxide metabolic process	6.86E-03
GO:0060415	muscle tissue morphogenesis	7.03E-03
GO:0030048	actin filament-based movement	7.05E-03
GO:0042632	cholesterol homeostasis	7.05E-03
GO:0055092	sterol homeostasis	7.05E-03
GO:0009314	response to radiation	7.09E-03
GO:0007141	male meiosis I	7.26E-03
GO:0015838	betaine transport	7.26E-03
GO:0015879	carnitine transport	7.26E-03
GO:0032371	regulation of sterol transport	7.26E-03
GO:0032374	regulation of cholesterol transport	7.26E-03
GO:0060601	lateral sprouting from an epithelium	7.26E-03
GO:0060041	retina development in camera-type eye	7.61E-03
GO:0006793	phosphorus metabolic process	7.69E-03
GO:0006796	phosphate-containing compound metabolic process	7.69E-03
GO:0015849	organic acid transport	7.71E-03
GO:0021826	substrate-independent telencephalic tangential migration	7.76E-03
GO:0021843	substrate-independent telencephalic tangential interneuron migration	7.76E-03
GO:0043116	negative regulation of vascular permeability	7.76E-03
GO:0046599	regulation of centriole replication	7.76E-03
GO:0048793	pronephros development	7.76E-03
GO:0050951	sensory perception of temperature stimulus	7.76E-03
GO:0043414	macromolecule methylation	8.04E-03
GO:0055008	cardiac muscle tissue morphogenesis	8.23E-03
GO:0031589	cell-substrate adhesion	8.26E-03
GO:0015697	quaternary ammonium group transport	8.40E-03
GO:0031057	negative regulation of histone modification	8.40E-03
GO:0051898	negative regulation of protein kinase B signaling cascade	8.40E-03
GO:0051493	regulation of cytoskeleton organization	8.42E-03
GO:0032886	regulation of microtubule-based process	8.59E-03
GO:0007169	transmembrane receptor protein tyrosine kinase signaling pathway	8.69E-03
GO:0051128	regulation of cellular component organization	8.94E-03
GO:0051347	positive regulation of transferase activity	9.11E-03
GO:0048812	neuron projection morphogenesis	9.38E-03

GO:0021987	cerebral cortex development	9.62E-03
GO:0044085	cellular component biogenesis	9.63E-03
GO:0048731	system development	9.77E-03

E

Neuronal Process	Accession	GO TERM	Robin	Canary
Development and Differentiation	GO:0048667	cell morphogenesis involved in neuron differentiation	1.50E-05	
	GO:0007399	nervous system development	8.05E-05	5.07E-03
	GO:0048666	neuron development	2.22E-04	2.81E-03
	GO:0030182	neuron differentiation	9.80E-04	
	GO:0007420	brain development	1.57E-03	
	GO:0007417	central nervous system development	3.78E-03	
	GO:0021772	olfactory bulb development	5.53E-03	
	GO:0021988	olfactory lobe development	5.53E-03	
	GO:0021537	telencephalon development	6.74E-03	
	GO:0021987	cerebral cortex development		9.62E-03
	GO:0051960	regulation of nervous system development		1.14E-03
Projection Development	GO:0048812	neuron projection morphogenesis	5.21E-05	9.38E-03
	GO:0031175	neuron projection development	2.03E-04	4.93E-03
	GO:0007409	axonogenesis	3.46E-04	
	GO:0007411	axon guidance	3.91E-04	
	GO:0008045	motor axon guidance	3.68E-03	
	GO:0010975	regulation of neuron projection development	7.34E-03	
	GO:0021952	central nervous system projection neuron axonogenesis		1.73E-03
	GO:0021955	central nervous system neuron axonogenesis		1.04E-03
	GO:0048841	regulation of axon extension involved in axon guidance		4.66E-03
Neurogenesis	GO:0048699	generation of neurons	7.18E-04	
	GO:0022008	neurogenesis	8.44E-04	
	GO:0022029	telencephalon cell migration	1.39E-03	1.54E-03
	GO:0021885	forebrain cell migration	1.76E-03	2.61E-03
	GO:0022028	tangential migration from the subventricular zone to the olfactory bulb	4.99E-03	4.25E-03
	GO:0021826	substrate-independent telencephalic tangential migration		7.76E-03
	GO:0021831	embryonic olfactory bulb interneuron precursor migration		5.08E-03
	GO:0021834	chemorepulsion involved in embryonic olfactory bulb interneuron precursor migration		1.43E-03
	GO:0021843	substrate-independent telencephalic tangential interneuron migration		7.76E-03
