# Supplementary material for: Regulatory mechanisms of testosterone-stimulated song in the sensorimotor nucleus HVC of female songbirds
Source: BMC Neurosci. 2014 Dec 2;15:128. doi: 10.1186/s12868-014-0128-0 (PMC4261767; doi:10.1186/s12868-014-0128-0)
Supplement: Additional file 8: Table S4. — Potential BDNF target genes. (A) Potential BDNF target genes that were up- or down-regulated after testosterone treatment in the HVC of female robins. An asterisk indicates response to testosterone in the HVC of female canaries. (B) Additional potential BDNF target genes not affected by testosterone in the HVC of female robins HVC. For references that indicate BDNF-dependent changes in other species see the end of the document. As expected from the multiple cellular functions of BDNF the bioinformatic analysis (GePS; IPA) of all potential BDNF target genes revealed consistently cellular processes including neurotransmission and synaptic plasticity, regulation of cell death/apoptosis as well as cell growth, differentiation and proliferation. [file 12868_2014_128_MOESM8_ESM.pdf]

A

Symbol	References	Fold change
FADS2	6	3.14
DNAJB5	10	2.33
SV2B*	38	2.30
GFRA1*	24	2.24
NPY*	1,2,6,7,10,21,22,24,26,29,35,36,37	1.97
RRAD*	24	1.95
DUSP6*	10,24	1.83
IFRD1*	24	1.73
NRN1	20,33	1.72
FOSL2*	1,17	1.71
NRP1*	24	1.71
GRIN2A*	5	1.68
HMGCR*	28	1.68
HOMER1*	13	1.68
TLL1*	38	-1.66
PRKG2*	31	-1.68
ZMIZ1*	33	-1.68
PER2*	10	-1.69
SCN2A*	8	-1.96

B

Symbol	References
ARC	1,10,23,34,39
ACVR1	24
ADAM19	24
ADCY8	24
ADRB2	24
AKAP1	31
AKR1A1	38
ANXA3	38
ANXA5	38
APOE	6
ARC	13
AREG	24
ARL4A	34
ARPP21	34
ATP6	31
ATP8	31
BAI2	24
BDNF	24
BHLHE40	10
BHLHE40	34

BTG2	24
CCK	7
CCND1	1
CCND2	10
CCNE1	38
CD9	38
CDKN1C	10
CPD	38
CREM	24
CXCR4	24
DCLK1	34
DTX3L	34
DUSP1	24
EGR1	1,10,24
EGR2	24
ELOVL6	10
ENO2	38
FBL	34
FGF10	38
FOS	1,12,17,24,38
FOSB	24
GABRB3	24
GAD1	19
GALR2	38
GAP43	17,38
GNAL	31
GNB2	38
GNG2	24
GRB14	10
GRIA1	4
GRIA2	4
GRIN1	5
GRIN2B	5
GRIP1	15
GSTK1	38
GSTP1	38
HIVEP1	10
HSD11B1	24
IER2	24
IGF2R	38
IGFBP6	38
IL17RA	24
INHBB	10
ITPKA	38
JUN	38
JUNB	17,24
JUND	38

KCNA1	1
KCNIP2	10
KCNJ5	38
KLF10	24,34
LHX5	38
LYPLA2	24
MAP2K3	24
MAP3K5	24
MAS1	24
MED16	34
MEF2C	18,33
MIF	38
MMP14	6
MMP2	27
MMP3	24
MMP9	27
MVD	28
MX1	31
NEFL	24
NPR1	31
NPTX1	24
NPTX2	34
NR4A1	24
NR4A2	24
NTRK2	16
NTSR2	38
NUSAP1	24
P4HA1	10
PAFAH1B2	24
PALMD	24
PDGFB	38
PDYN	7
PGAP2	38
PI4K2A	24
PICK1	15
PJA2	38
PLAT	9
PLDN	31
PMEPA1	34
PNOC	24
POR	38
PPP1R15B	24
PPP3R2	38
PRKAR1A	31
PRKCB	24
PSMA2	38
PSMB1	38

PSMB2	38
PSMB4	38
PTPN1	1
PTPN5	24
PTPRG	24
PTPRN	24
RAB2B	24
RAB3A	31
RAB3D	38
RGS16	24
RGS4	24
RGS7BP	31
RIOK3	31
RPS11	38
RYR2	24
SC5DL	24
SCG2	1,24,38
SCNN1B	1
SEPT9	24
SERPINB2	9,24
SFXN3	31
SGK1	24
SIAH2	24
SLC12A5	25
SLC18A1	38
SLC20A1	24
SLC2A3	3
SLC2A3	24
SLC6A1	19
SLC6A8	24
SLC9A1	38
SLC9A2	38
SNAP25	14,38
SPTBN4	24
SPTLC2	24
SRXN1	34
SST	6,7,10,21,22,24
SSTR2	24
SSTR4	1
ST8SIA3	24
STMN4	24
STX1A	14
SYNJ2	24
SYP	30
SYT1	30
TAC1	7,22,24
TAGLN3	24

TIMP2	38
TMSB10	10
TNC	38
TRH	11,32
TRIB2	10
TYMS	38
TYRO3	24
UNC5A	1
VAMP1	30
VGF	1,6
YWHAG	38
YWHAQ	38
YWHAZ	38

References used for table S4:

[1] Alder J, Thakker-Varia S, Bangasser DA, Kuroiwa M, Plummer MR, Shors TJ, Black IB: **Brain-derived neurotrophic factor-induced gene expression reveals novel actions of VGF in hippocampal synaptic plasticity.** *J Neurosci* 2003; 23:10800-10808.

[2] Barnea A, Roberts J: **Induction of functional and morphological expression of neuropeptide Y (NPY) in cortical cultures by brain-derived neurotrophic factor (BDNF): evidence for a requirement for extracellular-regulated kinase (ERK)-dependent and ERK-independent mechanisms.** *Brain Res* 2001, 919:57-69.

[3] Burkhalter J, Fiumelli H, Allaman I, Chatton JY, Martin JL: **Brain-derived neurotrophic factor stimulates energy metabolism in developing cortical neurons.** *J Neurosci* 2003, 23:8212-8220.

[4] Caldeira MV, Melo CV, Pereira DB, Carvalho RF, Carvalho AL, Duarte CB: **BDNF regulates the expression and traffic of NMDA receptors in cultured hippocampal neurons.** *Mol Cell Neurosci* 2007, 35:208-219.

[5] Caldeira MV, Melo CV, Pereira DB, Carvalho R, Correia SS, Backos DS, Carvalho AL, Esteban JA, Duarte CB: **Brain-derived neurotrophic factor regulates the expression and synaptic delivery of alpha-amino-3-hydroxy-5-methyl-4-isoxazole propionic acid receptor subunits in hippocampal neurons.** *J Biol Chem* 2007, 282:12619-12628.

[6] Cazzin C, Mion S, Caldara F, Rimland JM, Domenici E: **Microarray analysis of cultured rat hippocampal neurons treated with brain derived neurotrophic factor.** *Mol Biol Rep* 2011, 38:983-990.

[7] Croll SD, Wiegand SJ, Anderson KD, Lindsay RM, Nawa H: **Regulation of neuropeptides in adult rat forebrain by the neurotrophins BDNF and NGF.** *Eur J Neurosci* 1994, 6:1343-1353.

[8] Fanger GR, Jones JR, Maue RA: **Differential regulation of neuronal sodium channel expression by endogenous and exogenous tyrosine kinase receptors expressed in rat pheochromocytoma cells.** *J Neurosci* 1995, 15:202-213.

[9] Fiumelli H, Jabaudon D, Magistretti PJ, Martin JL: **BDNF stimulates expression, activity and release of tissue-type plasminogen activator in mouse cortical neurons.** *Eur J Neurosci* 1999, 11:1639-1646.

[10] Glorioso C, Sabatini M, Unger T, Hashimoto T, Monteggia LM, Lewis DA, Mirnics K: **Specificity and timing of neocortical transcriptome changes in response to**

BDNF gene ablation during embryogenesis or adulthood. *Mol Psychiatry* 2006, 11:633-648.

[11] Guerra-Crespo M, Ubieta R, Joseph-Bravo P, Charli JL, Pérez-Martínez L: **BDNF increases the early expression of TRH mRNA in fetal TrkB+ hypothalamic neurons in primary culture.** *Eur J Neurosci* 2001, 14:483-494.

[12] Ip NY, Li Y, Yancopoulos GD, Lindsay RM: **Cultured hippocampal neurons show responses to BDNF, NT-3, and NT-4, but not NGF.** *J Neurosci* 1993, 13:3394-3405.

[13] Ji Y, Lu Y, Yang F, Shen W, Tang TT, Feng L, Duan S, Lu B: **Acute and gradual increases in BDNF concentration elicit distinct signaling and functions in neurons.** *Nat Neurosci* 2010, 13:302-309.

[14] Johnston AN, Clements MP, Rose SP: **Role of brain-derived neurotrophic factor and presynaptic proteins in passive avoidance learning in day-old domestic chicks.** *Neuroscience* 1999, 88:1033-1042.

[15] Jourdi H, Iwakura Y, Narisawa-Saito M, Ibaraki K, Xiong H, Watanabe M, Hayashi Y, Takei N, Nawa H: **Brain-derived neurotrophic factor signal enhances and maintains the expression of AMPA receptor-associated PDZ proteins in developing cortical neurons.** *Dev Biol* 2003, 263:216-230.

[16] Kitagawa A, Nakayama T, Takenaga M, Matsumoto K, Tokura Y, Ohta Y, Ichinohe M, Yamaguchi Y, Suzuki N, Okano H, Igarashi R: **Lecithinized brain-derived**

neurotrophic factor promotes the differentiation of embryonic stem cells in vitro and in vivo. *Biochem Biophys Res Commun* 2005, 328:1051-1057.

[17] Koponen E, Lakso M, Castrén E: **Overexpression of the full-length neurotrophin receptor trkB regulates the expression of plasticity-related genes in mouse brain.** *Brain Res Mol Brain Res* 2004, 130:81-94.

[18] Liu L, Cavanaugh JE, Wang Y, Sakagami H, Mao Z, Xia Z: **ERK5 activation of MEF2-mediated gene expression plays a critical role in BDNF-promoted survival of developing but not mature cortical neurons.** *Proc Natl Acad Sci U S A* 2003, 100:8532-8537.

[19] Mizuno K, Carnahan J, Nawa H: **Brain-derived neurotrophic factor promotes differentiation of striatal GABAergic neurons.** *Dev Biol* 1994, 165:243-256.

[20] Naeve GS, Ramakrishnan M, Kramer R, Hevroni D, Citri Y, Theill LE: **Neuritin: a gene induced by neural activity and neurotrophins that promotes neuritogenesis.** *Proc Natl Acad Sci U S A* 1997, 94:2648-2653.

[21] Nawa H, Bessho Y, Carnahan J, Nakanishi S, Mizuno K: **Regulation of neuropeptide expression in cultured cerebral cortical neurons by brain-derived neurotrophic factor.** *J Neurochem* 1993, 60:772-775.

[22] Nawa H, Pelleymounter MA, Carnahan J: **Intraventricular administration of BDNF increases neuropeptide expression in newborn rat brain.** *J Neurosci* 1994, 14:3751-3765.

[23] Rao VR, Pintchovski SA, Chin J, Peebles CL, Mitra S, Finkbeiner S: **AMPA receptors regulate transcription of the plasticity-related immediate-early gene Arc.** *Nat Neurosci* 2006, 9:887-895.

[24] Ring RH, Alder J, Fennell M, Kouranova E, Black IB, Thakker-Varia S: **Transcriptional profiling of brain-derived-neurotrophic factor-induced neuronal plasticity: a novel role for nociceptin in hippocampal neurite outgrowth.** *J Neurobiol* 2006, 66:361-377.

[25] Rivera C, Li H, Thomas-Crusells J, Lahtinen H, Viitanen T, Nanobashvili A, Kokaia Z, Airaksinen MS, Voipio J, Kaila K, Saarma M: **BDNF-induced TrkB activation down-regulates the K+-Cl- cotransporter KCC2 and impairs neuronal Cl- extrusion.** *J Cell Biol* 2002, 159:747-752.

[26] Scharfman HE, Goodman JH, Sollas AL, Croll SD: **Spontaneous limbic seizures after intrahippocampal infusion of brain-derived neurotrophic factor.** *Exp Neurol* 2002, 174:201-214.

[27] Sun CY, Hu Y, Wang HF, He WJ, Wang YD, Wu T: **Brain-derived neurotrophic factor inducing angiogenesis through modulation of matrix-degrading proteases.** *Chin Med J (Engl)* 2006, 119:589-595.

[28] Suzuki S, Kiyosue K, Hazama S, Ogura A, Kashihara M, Hara T, Koshimizu H, Kojima M: **Brain-derived neurotrophic factor regulates cholesterol metabolism for synapse development.** *J Neurosci* 2007, 27:6417-6427.

[29] Takei N, Sasaoka K, Higuchi H, Endo Y, Hatanaka H: **BDNF increases the expression of neuropeptide Y mRNA and promotes differentiation/maturation of neuropeptide Y-positive cultured cortical neurons from embryonic and postnatal rats.** *Brain Res Mol Brain Res* 1996, 37:283-289.

[30] Tartaglia N, Du J, Tyler WJ, Neale E, Pozzo-Miller L, Lu B: **Protein synthesis-dependent and -independent regulation of hippocampal synapses by brain-derived neurotrophic factor.** *J Biol Chem* 2001, 276:37585-37593.

[31] Thakker-Varia S, Alder J, Crozier RA, Plummer MR, Black IB: **Rab3A is required for brain-derived neurotrophic factor-induced synaptic plasticity: transcriptional analysis at the population and single-cell levels.** *J Neurosci* 2001, 21:6782-6790.

[32] Ubieta R, Uribe RM, González JA, García-Vázquez A, Pérez-Monter C, Pérez-Martínez L, Joseph-Bravo P, Charli JL: **BDNF up-regulates pre-pro-TRH mRNA expression in the fetal/neonatal paraventricular nucleus of the hypothalamus. Properties of the transduction pathway.** *Brain Res* 2007, 1174:28-38.

[33] Wang Y, Liu L, Xia Z: **Brain-derived neurotrophic factor stimulates the transcriptional and neuroprotective activity of myocyte-enhancer factor 2C through an ERK1/2-RSK2 signaling cascade.** *J Neurochem* 2007, 102:957-966.

[34] Wibrand K, Messaoudi E, Håvik B, Steenslid V, Løvlie R, Steen VM, Bramham CR: **Identification of genes co-upregulated with Arc during BDNF-induced long-**

term potentiation in adult rat dentate gyrus in vivo. *Eur J Neurosci* 2006, 23:1501-1511.

[35] Williams AG, Hargreaves AC, Gunn-Moore FJ, Tavaré JM: **Stimulation of neuropeptide Y gene expression by brain-derived neurotrophic factor requires both the phospholipase Cgamma and Shc binding sites on its receptor, TrkB.** *Biochem J* 1998, 333:505-509.

[36] Wirth MJ, Obst K, Wahle P: **NT-4/5 and LIF, but not NT-3 and BDNF, promote NPY mRNA expression in cortical neurons in the absence of spontaneous bioelectrical activity.** *Eur J Neurosci* 1998, 10:1457-1464.

[37] Wirth MJ, Patz S, Wahle P: **Transcellular induction of neuropeptide Y expression by NT4 and BDNF.** *Proc Natl Acad Sci U S A* 2005, 102:3064-3069.

[38] Yang Y, Xie Y, Chai H, Fan M, Liu S, Liu H, Bruce I, Wu W: **Microarray analysis of gene expression patterns in adult spinal motoneurons after different types of axonal injuries.** *Brain Res* 2006, 1075:1-12.

[39] Ying SW, Futter M, Rosenblum K, Webber MJ, Hunt SP, Bliss TV, Bramham CR: **Brain-derived neurotrophic factor induces long-term potentiation in intact adult hippocampus: requirement for ERK activation coupled to CREB and upregulation of Arc synthesis.** *J Neurosci* 2002, 22:1532-1540.
